# Adaptation of the Coparenting Relationship Scale Questionnaire to Spanish Parents with Offspring

**DOI:** 10.3390/children11050535

**Published:** 2024-04-30

**Authors:** Dolores Seijo, Francisca Fariña, María Paula Fernández, Ramón Arce

**Affiliations:** 1 Forensic Psychology Unit, Department of Political Science and Sociology, Faculty of Psychology, University of Santiago de Compostela, 15782 Santiago de Compostela, Spain; mariadolores.seijo@usc.es (D.S.); ramon.arce@usc.es (R.A.); 2 Department AIPSE, University of Vigo, 36310 Vigo, Spain; francisca@uvigo.gal; 3 Department of Psychology, Oviedo University, Plaza Feijoo, 33003 Oviedo, Spain

**Keywords:** coparenting, coparenting relationship scale/CRS, engaged and separated/divorced parents, construct, convergent validity, discriminate validity, factorial invariance, coparental vitality

## Abstract

The scientific literature supports that practicing positive coparenting leads to the healthy development of children. Consequently, professional interest in parenting and coparenting has experienced significant growth, and evaluating coparenting is crucial in family psychology for establishing action protocols in clinical practice. An instrument highly regarded within the scientific community for evaluating coparenting dynamics is *The Coparenting Relationship Scale* (CRS). This research aims to achieve two objectives: first, to adapt the CRS for the Spanish population of both engaged and separated/divorced parents and to ascertain its reliability, validity, and factorial invariance psychometric properties; second, to assess the effectiveness of the total coparenting measure in categorizing sample participants. A cross-sectional non-experimental investigation was conducted to address these objectives. The first objective was answered by conducting an instrumental study, and the second by an exploratory study using classification techniques and a causal-comparative study using multivariate inferential methods. It was concluded that the model comprising 20 items across two factors, *Positive Coparenting* and *Negative Perception of Coparenting*, is the simplest and best fit for the Spanish parent sample; it is invariant regarding gender and marital status, and the measures derived from each factor demonstrate reliability and convergent and discriminant validity. The resulting questionnaire for Spanish parents is named CRS-S_Eg-S&D_. The *Coparental Vitality* measure calculated using the total weighted measure of CRS-S_Eg-S&D_ allows the sample of participants to be divided into three differentiated clusters called *Coparental Robustness*, *Moderate Coparenting*, and *Coparenting Rickets*.

## 1. Introduction

The scientific literature strongly supports the importance of coparenting in the proper development of children [1,2,3,4]. The exercise of parenting refers to the set of activities related to the care and education of children that each parent carries out, regardless of their legal or biological relationship [1,3,5,6]. Coparenting refers to how parents or caregivers relate to each other as such [7], irrespective of their sex. Today, the construct of coparenting has been decoupled from the gender role and is applied to all types of intact families [2].

When parents or parental figures agree to meet the needs of their children, they share the responsibilities of their upbringing by distributing the tasks related to their care, protection, and education, and they support each other in the parental role by facilitating and promoting positive interactions with their children (i.e., children must perceive that their parents agree on the rules they must follow; children must perceive that their parents address issues related to them together, support each other as parents, and avoid contradictions or competition between them in front of them). If the above description is true, they are exercising positive coparenting [1,4,8,9,10,11,12]. The coparenting relationship, although different from the couple relationship since it includes a romantic relationship between adults [9], is closely connected to it [7] and is of capital importance. Not in vain, the study of coparenting began by examining how the quality of the relationship that parents maintain when they have divorced influences their children [13,14,15,16]. It has been shown that when parents maintain a good relationship, their parental behaviors are positive, and the coparenting is harmonious; in addition to having advantages for themselves, it has clear advantages for their children, directly affecting their physical and psycho-emotional well-being [4,17,18,19,20,21,22], demonstrating that it is a powerful protective factor [2], for example, against addictions [4]. Often, when parents are separated or divorced, the consequences on their children are negative and contrary to these [2,4,13,14,15,16].

Thus, scientific and professional interest in parenting and coparenting has grown exponentially, and the evaluation of coparenting has become an essential issue in family psychology, both for research protocols and intervention in clinical practice [14]. This growing interest has led to the development of numerous self-report instruments aimed at capturing some of the multiple aspects that the construct entails [7,18], among them the *Parenting Alliance Inventory* [23], the *Coparenting Questionnaire* [24], and the *Coparenting Inventory for Parents and Adolescents* [25].

One of the instruments that has had the most significant impact and is best received by the scientific community is the Coparenting Relationship Scale (CRS) [26]. This scale can be used for families with children from infancy to adolescence. Based on family systems theory, Feinberg [9] proposes an ecological theoretical model of coparenting. (This model establishes the distribution between parents of the duties and responsibilities related to childcare. The parents agree on parenting between themselves and establish the roles of each one for joint management of the family, forming a coalition between the co-parental figures in front of the child. Each parent reinforces the other in their role as parents without any questioning or criticism between them). Thus, it places the coparenting relationship or the coparenting subsystem within the context of a broader family system where other social systems (e.g., economic context, religious context, and employment situation) are identified between which connections exist, where different family processes develop (it is considered that families are not static, but instead constantly changing due to the development processes of each family member), and where the main facets of the coparenting relationship are exercised [7,27,28]. New partners and children must be incorporated into the family naturally without posing an obstacle to the parental relationship.

The CRS questionnaire developed by Feinberg et al. [26] has been translated into multiple languages. The adequacy of its dimensional structure (long and short, 35 and 14 items, respectively) has been analyzed in different countries (Portugal, Sweden, France, Romania, and Spain/Spanish-speaking countries), and for samples with very *diverse particularities*, such as divorced parents (Portugal: [29]), father’s prenatal (Portugal: [30]), primiparous and multiparous fathers (Sweden: [31]), mothers (Portugal: [32]), minority and heterosexual people (Portugal: [33]), and parents of both sexes and all marital statuses in Portugal [34], France [35], Romania [36], and Spain/Spanish speakers [37].

Plá [37] translated and adapted the CRS questionnaire into Spanish in its two versions, long and short. However, it manifests important deficiencies in its approach and in the validation process, which we can summarize in five points. First, the sample consisted of 489 parents with at least one adolescent child between 11 and 18 years old. A total of 51.1% of the participants lived in Uruguay, and 48.9% lived in Spain. Furthermore, the Spanish participants were of 18 different nationalities. Assuming that the translation of the items had content that Spanish speakers of the 18 nationalities equally understood, it is very risky to believe that the dimensional structure found is valid for all of them because the different origins contain cultural differences, a widely documented aspect. In this sense, Bornstein [38] and Sun [4] have shown that the interaction between parents and children and cultural context may impact family functions, and Ronaghan et al. [7] insist that the country of the people can mediate the quality of parenting. Second, Feinberg et al. [26] found in the dimensional structure that the *Division of Labor* factor comprises only two items. Plá [37] added three more items, with common sense but without proven empirical support for this factor to be better represented. Third, Plá [37] tests the 7-factor model found by Feinberg et al. [26] in an exploratory way; however, they perform this task using Principal Component Analysis (PCA) instead of using Exploratory Factor Analysis (EFA). This aspect has been highly criticized by experts who unanimously conclude that PCA is not valid to study the dimensional structure of a questionnaire [39,40,41,42]. Fourth, Pla [37] does not show the descriptive statistics of the items referring to the central tendency, variability, and distribution, and this aspect is essential to evaluate the adequacy of the items. However, it does display the homogeneity index (HIc, the corrected correlation of each item with the test) and Cronbach’s alpha if the item is eliminated. It can be seen that item 5 has a value of 0.267, 0.221, and 0.297 in the long and short versions and in the belonging factor, respectively (the experts consider HIc < 0.30 [42,43,44] inadequate). It is also noted that the reliability of the questionnaire would increase significantly if this item were eliminated. This aspect has been observed in the adaptation of the questionnaire to other languages [29,32,33,34,36], and in all cases, it has been taken into account, and the item has been eliminated. However, it was not taken into account by Plá [37]. Fifth, in the request for seven factors (the same model found by Feinberg et al. [26]), two were underrepresented (appeared with two and three items, respectively), and one factor was overrepresented (appeared with eleven items). Consequently, since the seven factors of the CRS represented four theoretical domains (see Feinberg [9]), Plá confused domains with factors and requested four factors. Plá [37] finally concludes that the factors that make up the dimensional structure of the CRS-r are four, and demonized *Support received* or *Coparenting strength* (items 2, 3, 6, 10, 17, 19, 24, 25, 26, 27, 28, and 30 that correspond to the original scales *Coparenting Closeness*, *Coparenting Support* and *Coparenting Agreement*), *Exposure to Conflict* (for items 24–28, this factor remains fully equivalent to the factor of the original scale of the same name), *Agreement-non-sabotage* (items 8, 9, 11, 12, 13, 15, 16, 21, and 22, which correspond to the original scales *Coparenting Agreement* and *Coparenting Undermining*), and *Support given or solidarity* (items 1, 4, 5, 7, 14, 18, 20, 23, 31, 32, 33 that correspond to the original scales *Endorse Partner Parenting* and *Division of Labor*—it is necessary to remember that three more items were added to this factor). Item 29 of the original scale appeared unloaded and was the only item that was decided to be eliminated. However, in the final solution found by Plá [33], items 6 and 18 show a loading of <0.40, and items 18 and 33 are complex. Although both characteristics invalidate the relevance of the items [42,45,46], Plá [37] does not give importance to this aspect.

In this way, assuming that the items that make up the CRS include all the content of the coparenting construct that Feinberg et al. [26] identified, considering that “The advantages of this measure will also facilitate the assessment of the domains of coparenting in clinical practice, allowing intervention to capitalize on areas of strength and focus on improving areas of difficulty” [26] (p. 12), and considering that the adaptation and validation of the CRS scale to the Spanish population sample carried out by Plá [37] was not made appropriately, in this research, we propose two objectives. The first is to adapt *The Coparenting Relationship Scale* (CRS) [26] to the Spanish population of engaged parents (Eg) and separated or divorced parents (S&D) and to study its psychometric properties, that is, to determine its dimensionality, to test the hypothesis of factorial invariance as a function of sex and marital status, to study the reliability of the measure, and to examine evidence of validity. The second is to evaluate the strength of the total coparenting measure to classify the sample participants into different categories and thus deepen the knowledge of the coparenting construct.

A cross-sectional non-experimental investigation was carried out to respond to the stated objectives. The first objective was answered by conducting an instrumental study that followed the standards required for the construction, adaptation, and development of tests [47,48]. The second was answered by conducting an exploratory study using classification techniques and a causal-comparative study using multivariate inferential methods.

## 2. Materials and Methods

### 2.1. Participants

A total of 3155 parents submitted the answer booklet. Initially, the responses of 13 participants who had been widowed were eliminated (and for obvious reasons, their responses were not useful for the instrumental study because they were not coparenting). As a result, a total of 3142 participants were potentially useful to carry out the instrumental research: 2754 (87.65%) engaged parents (Eg, never separated or divorced, regardless of whether they are married or cohabiting together) [84.02% (n = 2314) and 15.97% (n = 440), respectively] and 388 (12.34%) separated or divorced (S&D) parents (188 S and 199 D).

First, a thorough study of the participants’ responses to the CRS items was conducted to clean the database [42,46] and preserve the sample of participants with useful responses to carry out the analysis. We began by identifying all *illegitimate cases* [42] (p. 85); these were those whose responses were useless for the intended objective, therefore lack value, and add substantial error variance to the analysis [42] (p. 85). Illegitimate cases were considered, such as those arising from non-responses to all scale items, for obvious reasons, and those arising from random response patterns likely to threaten the quality of the measure when carrying out a factor analysis. Random responses are a set of responses where individuals respond with little thought or reflection [49] due to a lack of preparation, reactivity to observation, lack of motivation to cooperate with the test, disinterest, or fatigue [49,50,51,52,53]. In this sense, it was established to reject the responses of those participants who did not respond to 40% or a greater percentage of the items in the CRS questionnaire. The argument is the following: This behavior could be due to a good intention (to respond later in a more reflective way, but never did so), but it could also be due to a lack of motivation and/or commitment to the research for which they had given their consent. On the other hand, of those participants whose response rate was complete, it was decided to eliminate those participants who maintained the same response level in more than 50% of the items (levels 3 and 6 were the most selected).

Thus, 218 participants did not respond to any item on the scale, 271 left the responses to more than 40% of the items blank, and 102 gave the same response to more than 50% of the items. In total, 591 participants submitted the booklet with little or no commitment to the research. We considered the pattern of their responses to be random; therefore, their responses were useless for the analysis. Thus, the responses of 2551 people are potentially useful for data analysis.

Among these, 124 people left some items empty (between 1 and 5 items), possibly due to random (MAR) or non-random (NMAR) causes. They were also eliminated because they represent a percentage of less than 5% (4.86%), and their elimination does not threaten the validity of the statistical and substantive conclusions of the analysis results [54]. As a result, the sample finally consisted of 2427 parents.

### 2.2. Sociodemographic Characteristics of the Sample

Regarding marital status, 90.1% (n = 2187) are Eg and 9.9% (n = 240) are D&S (technically, distributed in the same proportion, 4.9%, n = 119, are separated and 5%, n = 121, are divorced). The number of women is four times greater than that of men [women are 80.4% (n = 1951) of the sample, and men are 19.6% (n = 475)], and the mean age is 40.42 years [SD = 5.67; range 1–66 years; bias = −0.291 and kurtosis = 1.27].

On the other hand, 96.4% do not have other children from previous relationships. Only 3.6% (n = 87) have children from previous relationships [2.7% (n = 66) have one more child from earlier relationships, 0.8% (n = 19) report having two children from previous relationships, and only 2 people have 3 children from previous relationships]. Regarding the age of those children they have with previous partners, for people who have a child, the age of these children is between 1–33 years (M = 17.74; SD = 6.39). Of those who have two children, the first is in the range of 10–29 years (M = 19.65; SD = 5.88), and the second is in the range of 1–25 years (M = 15.68; SD = 6.33). Those who have three children (which are only two people) have had children after a long time because the first is in a range of 21–24 years (M = 22.5; SD = 2.12), the second is in a range of 21–23 years (M = 22; SD = 1.40), and the third is in the range of 19–27 years (M = 23; SD = 5.65).

Regarding the current family at the time they participated in this research, 89.4% (n = 2154) is a primary family (both are biological or adoptive parents), 3.3% (n = 80) is a reconstructed family (at home, there is a stepfather/stepmother), 3.7% (n = 89) is a family of single parents, and 3.5% (n = 84) state that they are in a different situation from the previous ones. Regarding the number of children living at home, the most frequent situation is where 2 children live together, 55% (n = 1336), followed by where only one child lives together, 33.8% (n = 820). Far removed from these two predominant conditions, three children live together in 8.9% (n = 217) of the cases; in 30 homes (1.2%), there are four children, but there is also the case where 5, 6, and 7 children live together, which total 9 homes (0.3%). That is, their child, about whom they have responded in the booklet, is an only child or is the only child in the house 33.8% of the time (n = 820), lives with a sibling 55% of the time, and are among 3 or more children at home 11.2% of the time.

Regarding the children under 12 years of age for whom the participants responded to the items in the booklet, 50.9% are male (n = 1236), and 48.4% are female (n = 1174). Only 3.8% are 12 (n = 93), and 2-year-olds are 4.6% (n = 108). Thus, it can be considered that between 2–12 years, all ages are represented. The average age is 7.23 years [SD = 2.80; range 1–12 years]. Age is non-normally distributed [bias = −0.119 and kurtosis 1.039; K-S = 0.056, *df* = 3120, *p* = 0.000; P_25_ = 5, P_75_ = 10, P_50_ = 7).

The participants who were eliminated from the instrumental study (the 591 participants who submitted the booklet with little or no commitment to the research and the 124 we estimated who left items unanswered for MAR or NMAR reasons) did not differ in the sociodemographic characteristics from the 2427 parents who were part of the study.

### 2.3. Procedure

The researchers contacted 130 elementary schools (primary schools) in Spain, and 73 agreed to collaborate (characteristics of the schools, location, rural vs. urban, size, and participation are shown in Section S5 of the Supplementary Materials). A conference was organized in each of the 73 elementary schools aimed at all the students’ families. All attendees were informed that research was being carried out to expand knowledge about parenting and coparenting, and they were shown the content of the answer booklet. All fathers and mothers with sons and daughters between 2 and 12 years old were invited to participate.

To ensure the maximum possible participation and response rate and avoid acquiescence in the response, extreme precautions were taken throughout the data collection process [54,55]. In this sense, the participants were warned that paying attention to the questions and answering honestly was necessary, only referring to the youngest of their children. Furthermore, due to the booklet length, they were informed that they would have one month to respond. They were also told that during that time, they could contact the project researchers to resolve any questions, and a telephone number and email address were provided. All attendees were informed that the school management team would send four reminders to avoid oblivion (one weekly reminder for four weeks in the school’s usual way of communicating with students’ families).

People who gave informed consent received the response booklet and an opaque envelope. To guarantee anonymity and discretion, an ad hoc mailbox was set up in each school where they had to insert the anonymous answer booklet inside the opaque envelope when they had completed it. The research respected all laws on protecting personal data and had permission from the Bioethics Committee of the University of Santiago de Compostela.

### 2.4. Measurements

The Coparenting Relationship Scale (CRS) [26]. The questionnaire consists of 35 items, divided into 7 dimensions of coparenting related to the four domains of Feinberg’s model [9] as follows:

1. The domain of coparenting agreement with a subscale of the same name, Coparenting agreement (4 items);
2. The coparenting support/undermining domain was represented by 3 subscales, Coparenting Support (6 items), Endorsement of Partner’s Parenting (7 items), and Coparenting Undermining (6 items);
3. The domain management of family relationships was assessed with the subscale Exposure to Conflict (5 items);
4. The domain division of childrearing work was made up of the subscale called Division of Labor (2 items).

Finally, Feinberg et al. [26] created a 5-item subscale measuring the degree to which coparenting enhanced intimacy and strengthened the couple’s relationship, which they called Coparenting Closeness (5 items).

Feinberg et al. [26] concluded that “The overall Coparenting Relationship Scale demonstrated excellent internal consistency, with Cronbach’s alphas ranging from 0.91 to 0.94 across gender and data collection time” [26] (p. 8).

All items have a 6-point response scale ranging from 0 “not true of us” to 6 “very true of us”, except for the Exposure to Conflict subscale, where responses ranged from 0 “never” to 6 “very often”. For correction, the mean score for the items of each of the subscales is calculated (14 items are inverse, see Sections S1 and S2, Table S1 of the Supplementary Materials). Example items are *I believe my partner is a good parent*; *My partner and I have the same goals for our child*; *My partner does not trust my abilities as a parent*.

Parenting and Family Adjustment Scales (PAFAS). Sanders et al. [56] developed and adapted to Spanish by Fariña et al. [57], resulting in a valid, reliable, brief, and comprehensive measure to evaluate Spanish parents’ parenting styles and family adjustment. It consists of 20 items distributed in two subscales and five factors. The Parenting subscale is made up of the factors of Coercive parenting (5 items), Positive stimulation (3 items), and Maternal/Paternal filial relationships (4 items), and the Family Adjustment subscale is made up of the factors of Parental adjustment (4 items) and Adjustment family (4 items). The reliability coefficient was calculated using the coefficient H; in both subscales, it was 0.96. Participants respond to the extent that each statement is correct for their situation on a Likert-type scale, with 4 response alternatives (0 “never”, 1 “rarely/sometimes”, 2 “quite a few/many times”, 3 “most of the time/always”). Example items in the Parenting subscale include *I shout or get angry with my child when they Misbehave*; *I give my child a treat, reward or fun activity for behaving well*; and *I give my child attention (e.g., a hug, wink, smile or kiss) when they behave well*. Example items in the Family Adjustment subscale include *I cope with the emotional demands of being a parent*; *I work as a team with my partner in parenting*; and *I feel happy*.

Parental Efficacy Scale (CAPES). It was built by Morawska et al. [58] and adapted with sound psychometric properties for the Spanish population by Seijo et al. [59]. The scale comprises 25 items grouped into two factors: Child’s Competencies (10 items) and Behavioral and Emotional Problems (15 items). The reliability coefficient calculated using Cronbach’s alpha was 0.94 and 0.84 in the respective scales. Participants respond to each item on a 4-point Likert-type scale, from 0, “not true at all”, to 3, “true most of the time”, depending on how true the statement was for their child in the past 4 weeks. Items are summed to yield a total intensity score (CAPES intensity scale) composed of a behavioral score and an emotional maladjustment score. Higher scores indicate higher levels of problems. Example items in the Child’s Competencies factor include *Can keep busy without constant adult attention*; *Does what they are told to do by adults*; and *Talks about their views, ideas and needs appropriately*. Example items in the Behavioral and Emotional Problems include *Gets upset or angry when they don’t get their own way*; *Loses their temper*; and *Takes too long getting dressed*.

### 2.5. Data Analysis

We can summarize the axes that determine the data analysis in the process of adapting the CRS questionnaire [26] to the Spanish population of engaged parents (Eg) and separated or divorced parents (S&D) into two.

In the first axis, the response booklet indicated that items 31–35 (corresponding to the *Exposure to Conflict* scale) would only be answered if a couple was broken up, that is, separated or divorced. This was performed for four reasons. The first is because the same authors who constructed the questionnaire write, “However, we believe that coparenting relations may differ from one child to the next, and thus recommend that some subscales (e.g., exposure to conflict) be administered regarding each child separately” [26] (p. 12). Therefore, we understand that the authors contemplate a certain freedom in how these items are considered. The second is because the items that represent the *Exposure to Conflict* factor are ordered consecutively and not presented in random order among the set of scale items, as are the rest. Due to the influence that the order of presentation of the items has on the response of the participants, as highlighted in the previous section [60,61], we consider that it is possible that the response to the first item conditions the response to the others, even from the rest of the items (because there is no control over the order in which participants respond to the items), causing an unwanted response pattern in the questionnaire. Third, Feinberg et al. [26] found a mean value and a standard deviation less than 1 in this factor. This result indicates that the items that make up the factor are not discriminative in the population of parents to which the sample belongs [43]. Pinto et al. [30] decided to eliminate these items to find a good fit in the model, possibly for the same reason. The common denominator of these two investigations was the sample. In Feinberg et al. [26], the sample was heterosexual couples who were expecting their first child at the time of recruitment, and in Pinto et al. [30], the sample was the father’s prenatal., Fourth, because Feinberg et al. [26] and all the research above that has adapted and validated the CRS questionnaire to other countries and/or other *sample particularities* have found a correlation ≥ 0.60 between the *Exposure to Conflict* and *Coparenting Undermining* scales, therefore, we estimate that the magnitude of the conflict that may exist between members who have children in common, whether they were together or separated or divorced, can also be captured with the items of the *Coparenting Undermining scale*, thus making the duration of the questionnaire shorter. Therefore, in this research, the 6-factor model is studied in a 30-item test, and we call this initial model CRS^6F^.

As explained in the description of the procedure in Section 2.2, this research is part of a more extensive investigation on coparenting and parenting, and the added value of the *Exposure to Conflict* factor in the evaluation of coparenting exclusively in divorced and/or separated is the subject of future research.

In the second axis, Feinberg et al. end the article as follows: “We hope that this measure, or future refinements of it, will be useful for examining family relationships across various contexts. We look forward to further inquiries examining the reliability and validity of this measure in an array of families, with diverse sociodemographic backgrounds, levels of risk, and stages of family development” [26] (p. 12). This research has these words as its starting point, and what is pursued in achieving the two objectives stated above is to select those items from the CRS^6F^ that are useful and valid to evaluate coparenting both in parents who live together and in parents who do not live together and have separated or divorced, and in the same way for men and women. That is, we intend to select items that allow us to evaluate coparenting and that are invariant depending on the marital status and sex of the parent to study the coparenting relationship in depth. To this end, a core part of this research is the development of an exhaustive Exploratory Factor Analysis (before testing a theoretical model through Confirmatory Factor Analysis), where the relevance of the items is carefully examined in the different subsamples of parents and where emphasis is placed on the analysis of the replication of the results, as detailed below.

Data analysis was organized into three main blocks. 

#### 2.5.1. Block of Analysis 1: Analysis of the Factor Structure of the Questionnaire, Determination of Its Dimensionality, and Study of the Reliability of the Measure

The process of assessing the dimensionality structure of *The Coparenting Relationship Scale* (Feinberg et al. [26]) began by assessing whether the 6-factor model in a 30-item test (model CRS^6F^) was valid in the Spanish population of engaged parents (Eg) and separated or divorced parents (S&D). This was conducted in two ways: by a Semiconfirmatory Factor Analysis (sCFA) using Procrustean rotations against a target matrix [62] and by the Confirmatory Factor Analysis (CFA). Both methods converge in that the original model does not fit the data. Thus, a study was conducted to determine which dimensional structures are appropriate and their compositions.

Following the required procedure for cross-validation [63], we used an *internal replicability analysis*, and the sample was randomly divided into two independent samples, taking extreme care so that the participants in both subsamples were balanced according to the variables sex and marital status.

With the calibration sample (n = 1239), a successive Exploratory Factor Analysis (EFA) was performed, and the most suitable items were selected. Previously, a descriptive study of the items in each subgroup defined by sex and marital status was carried out (see Section S2 of the Supplementary Materials), and it was determined to eliminate all inappropriate items. Experts consider items with the corrected homogeneity index (HIc) < 0.25 [43], skewness > 3 and kurtosis > 6 [64,65], standard deviation less than 1, and items that have a mean value close to the maximum or minimum value of the item response [39,43]. Next, in successive EFAs, the items without loading, the complex items, and the items loading less than 0.40 were eliminated one by one and in this order [66] until a simple, clear, and interpretable solution was obtained. The model best fitted by EFA was called model M1 and was composed of 21 items dimensioned in 2 correlated factors.

The sCFA was performed using the FACTOR program (V.11.04.02) [67], which examines the model fit based on the Root Mean Square Deviation (RMSD) [68]. If the RMSD < 0.05, the misfit is trivial; between 0.05 and 0.10, it is moderate, and if the RMSD > 0.10, the misfit is substantial [69,70]. The descriptive study of the items was carried out using IBM SPSS 27. The EFA was carried out with FACTOR and with JASP (V.0.14.1.0) (both provide complementary information), and CFA (described later) was carried out with JASP.

Because the items are ordinals and some items showed skewness and/or kurtosis values significantly far from normality, the polychoric correlation matrix was used in all the EFA and CFA models tested [62,71]. The ordinal nature of the items also determined the estimation method used. For all EFA models, the estimation procedure was Minimum Residuals in JASP and Robust Unweighted Least Squares (RULS) in FACTOR (both procedures are equivalent [72]). The number of factors to be retained was determined by taking into account the result of the optimal implementation of the Parallel Analysis (PA, [73]) and by considering eigenvalues above 1 (Kaiser’s criterion) and the Scree Test. For the correlation between the factors to be expressed in their full magnitude, the direct solution was obliquely rotated using Promin robust rotation in FACTOR [74] and oblimin rotation in JASP. The models were evaluated with the Root Mean Square Error of Approximation (RMSEA), Tucker–Lewis index (TLI), and BIC. Satisfactory reference values are RMSEA ≤ 0.06, TLI ≥ 0.95 [66,75], and a lower BIC. The simplicity of the model was assessed using the S index [76].

Next, in the calibration sample, three aspects were evaluated. First, it was assessed whether the factor solution of M1 approaches unidimensionality. This was considered using the indices UniCo, ECV, and MIREAL. Data can be treated as essentially unidimensional when UniCo > 0.95, ECV > 0.85 or MIREAL < 0.30 [77]. Second, it was evaluated whether a second-order factor could exist. The Added-Value analysis [78] will allow us to decide by observing the mean squared error reduction (PRMSE) if a second-order factor better defines the dimensionality. Third, the strength of construct replication by the H index [77] was evaluated. High H values (>0.80) suggest a well-defined latent variable, which is more likely to be stable across studies. In contrast, low H values indicate a poorly defined latent variable, which is expected to change across studies.

Before performing the CFA in the validation sample (n = 1188), EFA also examined whether the items would be assigned to the same factors and whether the items’ factor loadings would have an equivalent magnitude in both samples. The difference between the standardized factor loadings was calculated and squared for each item. The squared difference > 0.04 indicates that the factor loadings are volatile, and the construct will not be replicated in other samples [42]. It was verified that all items were positioned the same way as in the calibration sample and that no item was volatile. Thus, once the replication was confirmed in the validation sample, Model M1 was tested by CFA, showing a satisfactory fit. However, it was decided to eliminate one item because it had R^2^ < 0.20 and a standardized factor loading < 0.50. This most parsimonious and best-fitting model is Model M2, which was tested to assess factorial invariance.

The CFA used the Diagonally Weighted Least Squares with Mean and Variance corrected (WLSMV) [79] estimation method. Based on the modification indices, the correlation between the errors was left free. The fit of the model was examined by 4 indices: RMSEA, Standardized Root Mean Square of Residuals (SRMR), the comparative fit measure concerning the null model of independence (Confirmatory Fit Index, CFI), and the χ^2^/*df* ratio. Satisfactory reference values of the latter three indices were SRMR < 0.08, CFI ≥ 0.95 [75,80], and χ^2^/*gl* < 3 [81]. Next, the multi-group CFA according to sex groups and marital status was performed. The deviation of the metric, scalar and strict invariance models from the configurational invariance model was examined based on the magnitude of changes in fit indices in CFI, RMSR and RMSEA [82,83]. Chen [82] recommend using CFI for the invariance evaluation first, supplemented by RMSEA and SRMR. A change of −0.010 or more in the CFI combined with changes in RMSEA of 0.015 and SRMR of 0.03 (for metric invariance) or 0.015 (for scalar or residual invariance) was used as an indication of non-invariance. The internal structure analysis was concluded by examining composite reliability (CR) [66].

The reliability of the measure of the resulting scale, CRS-S_Eg-S&D_, was then estimated by analyzing internal consistency using Cronbach’s standardized alpha and McDonald’s ordinal omega. Values greater than 0.70 were considered acceptable [66].

#### 2.5.2. Analysis Block 2: Study of the Evidence of Validity

Convergent and discriminant validity were analyzed using different estimators. On the one hand, it was examined on the basis of the correlation between the factors of the CRS-S_Eg-S&D_ and the factors of the PAFAS [57] and CAPES [59] questionnaires. This was performed independently for the total sample and for each marital status. Values of r ≥ 0.20, r ≥ 0.50 and r ≥ 0.80 express a weak, moderate and strong correlation, respectively [84]. On the other hand, convergent validity was examined by jointly analyzing the average variance extracted (AVE) and composite reliability (CR) derived from the CFA [85], and considering the standardized factor loadings [66,86].

Discriminant validity existed if the square root of AVE was greater than the square between the correlations of the latent factors [85], and convergent validity if AVE > 0.5.

#### 2.5.3. Analysis Block 3: Evaluation of the Strength of the Total Coparenting Measure Calculated Using CRS-S_Eg-S&D_ to Classify the Sample Participants into Different Categories

A two-stage cluster analysis was carried out, randomizing the order of the participants on 20 occasions as a sensitivity analysis and as an evaluation of the replicability of the result. The quality of clusters was considered poor, sufficient, or good based on the work developed by Kaufman and Rousseeuw [87]. MANOVA was then performed to determine how different the resulting clusters were and how different they were in each of the two factors that make up the total measure of the CRS-S_Eg-S&D_. The analysis was completed by performing a stepwise discriminant analysis [88,89] to examine which dependent variable (Factor 1 or Factor 2) had the most strength to differentiate between the clusters, or if the discrimination strength resided in a combination of both, providing special attention to the standardized coefficients and the magnitude and sign of the centroids. Finally, in the clusters identified based on the total measure of the CRS-S_Eg-S&D_, the measure of the two dimensions in each group resulting from the combination Sex x Marital Status was represented graphically.

The correlation observed between the empirical scores of the 2 factors was r = 0.576 (*p* < 0.001). Therefore, it constituted an appropriate condition (0.30 < r < 0.80) to use MANOVA [90,91] and control the Type I error that could accumulate if independent ANOVAs were performed instead [66,92]. Because the covariance matrices were heterogeneous (Box’s M = 181.66; *p* = < 0.001), the result was examined using Wilks’ Lambda statistic [93], and resampling was used to estimate the parameters [94]. The level of significance was α = 0.05, and the reference values 1 − β > 0.80, and partial eta squared (η^2^) of 0.01, 0.06, and 0.14 represented small, medium, and large, respectively [95,96].

## 3. Results

### 3.1. Evidence of Validity Based on the Internal Structure and Reliability of the Scale Score

The results are shown in Table 1 and Table 2.

The model found by Feinberg et al. [26] of 30 items and six factors, Model CRS^6F^, does not fit the data from the sample of Spanish engaged parents (Eg) and separated or divorced parents (S&D). Both methods, sCFA and CFA, allow us to conclude the same result. In the sCFA, the RMSD values for the six subscales were 0.207, 0.148, 0.128, 0.163, 0.188, and 0.086, indicating a moderate mismatch for the Coparenting Closeness subscale (0.086), and a substantial mismatch for the remaining five subscales, with the total mean mismatch being 0.158. The initial CFA showed a very unsatisfactory fit only in χ^2^/*df* (χ^2^/*df* = 7.62; CFI [TLI] = 0.968 [0.964]; SRMR = 0.071 and RMSEA = 0.052) (see Table 2), and although all standardized factor loadings were statistically significant, the loadings of the items *The stress of being parents has distanced us as a couple* (item 28), *The other parent does not trust my abilities as a parent* (item 13), and *The other parent likes to play with the child but leaves the unpleasant work for me* (item 5), belonging to the subscales *Coparenting Closeness*, *Coparenting Undermining* and *The Division of Labor*, respectively, were lower than what experts [66,80] consider reasonable (0.410, 0.451, and 0.313, in the order cited). In addition, the R^2^ values of the items *The stress of parenthood has caused my partner and me to grow* (item 28), *My partner doesn’t like to be bothered by our child* (item 29), *My partner does not trust my abilities as a parent* (item 13), *My partner likes to play with our child and then leave dirty work to me* (item 5), and *El otro progenitor no asume la resposabilidad como padre/madre* (item 20), belonging to the subscales *Coparenting Closeness*, *Endorsement of Partner Parenting*, *Coparenting Undermining* and the *Division of Labor* were also notably low (0.168, 0.250, 0.204, 0.089, and 0.276, respectively). Even so, the internal consistency examined using the standardized Cronbach’s alpha of the subscales *Coparenting Agreement*, *Coparenting Closeness*, *Coparenting Support*, *Endorsement of Partner Parenting*, *Coparenting Undermining* and *The Division of Labor*, and the total scale, was, respectively, 0.764, 0.780, 0.916, 0.866, 0.803, 0.282, 0.943. See the wording of the CRS items in both languages, English and Spanish, in Section S1 of the Supplementary Materials.

A modeling process was then initiated with the calibration sample (n = 1239) through successive EFAs. In the application of the CRS^6F^ Model found by Feinberg et al. [26], it was found that the dimensional structure was complex (S = 0.212) and the CFI was unsatisfactory (0.881) (see Table 2) because some items were represented in more than one factor, other items were not in none, and some factors were not represented (see Section S3 of the Supplementary Materials, Table S5). With resounding unity, PA, the Scree Test, and Kaiser’s criterion indicated that two factors were appropriate to represent the dimensionality of the set of 30 items. Thus, first of all, based on the descriptive statistics of the items in the calibration sample (observed according to sex and marital status), items 1, 4, 7, 21, 22, and 5 were eliminated because they were inappropriate (the first five items, because they showed inadequate descriptive statistics to be part of an FA in the Eg subsample, male and female, and item 5 because the four subsamples showed a HIc value < 0.30; see Tables S1–S4 in Section S2 of the Supplementary Materials). Once the above analysis was performed, modeling using the EFA began, and items 20, 28, and 13 were eliminated (items 20 and 28 were then eliminated because they had a factor loading < 0.40; once the previous two were eliminated, item 13 showed no loading and was also eliminated). As a result, a total of nine items were eliminated. Thus, Model M1 by EFA sized with two factors consisting of a total of 21 items (F1 and F2 have 14 and 7 items, respectively) has a very satisfactory fit (BIC = −0.536; CFI = 0.912; RMSEA = 0.071; S = 0.999) (see Table 2). The uniqueness (<0.70), the communality (>0.50), and the standardized loadings factorials (>0.50) are appropriate for all items (see Table 1), and the model explains 63.89% of the variance (0.526% and 0.112%, F1 and F2, respectively). The data adequacy examined using the KMO sphericity and Bartlett’s tests was satisfactory in all models examined.

On the other hand, the UniCo = 0.916 and ECV = 0.821 indexes show that the structure of the M1 model moves away from unidimensionality. The Added-Value analysis allows us to conclude that the model with two primary factors fits significantly better (PRMSE = 0.901 [0.888–0.910] and 0.967 [0.963–0.970] for F1 and F2, respectively) than a model with a second-order factor (PRMSE = 0.355 and 0.703 for F1 and F2, respectively). The H index indicated that the latent variables in F1 and F2 were well defined (latent H index = 0.901 and 0.967, for F1 and F2, respectively), and the squares of the difference between the standardized factor loadings of the EFA of M1 in the calibration and validation samples were less than 0.04, demonstrating that the dimensional structure was replicable in the same factors and the same items (see Table 1). Additionally, the internal consistency was very satisfactory (alpha = 0.943, 0.839, and 0.935 for F1, F2, and the entire scale, respectively).

Therefore, it was concluded that Model M1 sized by EFA is the simplest and the best-adjusted model. It has been replicated in the validation sample, the latent variables F1 and F2 are well defined, and the measures of F1 and F2 in the Spanish sample of Eg and S&D are reliable.

The CFA with the validation sample (n = 1188) corroborated a satisfactory fit of the M1 Model (χ^2^/*df* = 1.08; CFI [TLI] = 0.999 [0.999]; SRMR = 0.035 and RMSEA = 0.008) (see Table 2). However, it was decided to eliminate item 16 because it showed an R^2^ lower than 0.30 (R^2^ = 0.234) and a standardized factor loading lower than 0.50 (F. loading = 0.484). The resulting Model, the M2 Model, has an even more satisfactory fit than M1 (χ^2^/*df* = 1.04; CFI [TLI] = 1 [1]; SRMR = 0.034 and RMSEA = 0.006) (see Table 2). The composite reliability is excellent, 0.946 and 0.826 for factors F1 and F2, respectively.

The factorial invariance of M2 as a function of sex and marital status was then tested. Based on the fit indexes χ^2^/*df*, CFI, and RMSEA, and based on the fact that the magnitude of the changes in absolute values of CFI, RMSR, and RMSE [82,96] does not exceed the recommended limits [82,83,96], it could be concluded that there is strong configurational, metric, scalar and strict invariance for males and females and engaged parents and separated or divorced parents (see Table 2), and therefore, the items measure the same dimensions with the same structure, regardless of sex and marital status. This property is a prerequisite so that the empirical scores for each factor can be compared and interpreted validly [97].

In M2, the internal consistency evaluated by Cronbach’s alpha test (0.943 and 0.829, for F1 and F2, respectively) and by McDonald’s ordinal omega (0.943, 0.831 for F1 and F2, respectively) was adequate.

It is thus concluded that M2 sized with two factors consisting of a total of 20 items is the simplest, best adjusted model for the sample of Spanish parents, it is invariant according to sex and the marital status, and the measure derived from each of its factors is reliable. The new questionnaire for Spanish parents is called CRS-S_Eg-S&D_. The resulting factors were defined as *Positive Coparenting* (F1, 14 items) and *Negative Perception of Coparenting* (F2, 6 items), and both measure the construct that we call *Coparenting Vitality*.

### 3.2. Evidence for Convergent and Discriminate Validity

Some results are shown in Table 3. See Section S3 of the Supplementary Materials (Tables S7 and S8).

*On the basis of the relationship between the factors of the* CRS-S_Eg-S&D_. The correlation in the total sample between the direct scores of the factors F1 (*Positive Coparenting*) and F2 (*Negative Perception of Coparenting*) is r = 0.512 (*p* < 0.001), and this relationship is maintained in both samples of parents (Eg and S&D), although it is slightly lower with respect to that calculated in the total sample because the variability is less (see Table 3).

*Based on the relationship between the empirical scores of the* CRS-S_Eg-S&D_ *factors with the factors of the PAFAS and CAPES*. Table 3 shows that in the sample of Eg parents, there is a statistically significant correlation between F1 CRS-S with F22 PF and PF-T (r = 0.601 and r = 0.510, respectively) and also between CRS-S-T with F22 PF and PF-T (r = 0.599 and r = 0.516, respectively). On the other hand, in the subsample of S&D parents, there is only a statistically significant relationship between CRS-S-T and F22 PF (r = 0.522). The most notable thing is that in the S&D subsample, the link between F1 CRS-S and F22 PF disappears, which does appear in the Eg subsample. The magnitude of the correlation is an indicator of the existence of convergent validity, as expected. The divergence between what happens in the S&D and Eg subsamples is an indicator that F1 CRS-S is capable of identifying differences between S&D and Eg, and this was an objective of this research. These should be studied in future research because they are indicators of a solid practical implication.

It is also notable that, in the same way, in both subsamples, F2 CRS-S is not significantly related to any of the PAFAS or CAPES factors. This aspect is very relevant and also requires further investigation. A priori means that the PAFAS questionnaire does not evaluate a Negative Perception of Coparenting and that a Negative Perception of Coparenting is not related to the behavior of the children assessed through the CAPES.

*On the basis of the relationship between the latent variables*. The correlation between the latent factors is r_l_ = 0.563 (*p* < 0.001). AVE from F1 and F2 are 0.5627 and 0.444, respectively, indicating high convergent validity in F1 [85] and marginal convergent validity in F2. Anyway, given that CR is always greater than 0.6 for all latent constructs, convergent validity could also be concluded for F2 [85] (p. 46). Furthermore, in all cases, the square root of AVE is greater than the square between the correlations of the latent factors. Therefore, it can be concluded that discriminant validity exists too [85].

### 3.3. Evaluation of the Strength of the Total Coparenting Measure Calculated Using CRS-S_Eg-S&D_ to Classify the Sample Participants into Different Categories

In all published works that use the CRS questionnaire [26], the calculation of the mean for each factor is proposed, and the calculation of the total mean of the set of items is proposed as an estimate of a total measure of coparenting. However, the factors found by Feinberg et al. [26] have a different number of items (there are factors with two, four, five, six, and seven items), and thus the factors that have more items have a greater weight in the total sum and therefore in the total average. Furthermore, a total average dissolves the particular contribution of each factor. In this research, we propose calculating the measure of each factor as a weighted adjusted measure in the following way. The sum of the items of each dimension is divided by the total sum possible in the factor. Because the CRS questionnaire adapted to the Spanish population comprises two factors, the previous result is multiplied by 0.5. Then, the value obtained from the two factors is added algebraically. The maximum possible measurement of each of the two factors will be 0.5, and the maximum value of the total measurement in CRS-S_Eg-S&D_ will be one. In this way, the measurement of both factors can be compared in different circumstances, and it will be possible to observe which factor contributes the most to the total measurement. However, when the measurements of each factor are compared, it can be performed with the average or with the result of this calculation. The results of the statistical analyses will be identical. However, when the total score for CRS-S_Eg-S&D_ is examined, it should be performed in this way that we propose to attribute equal weight to the two dimensions that make up the construct and not dissolve the variability contained in the dimensions it comprises.

**Figure 1:**
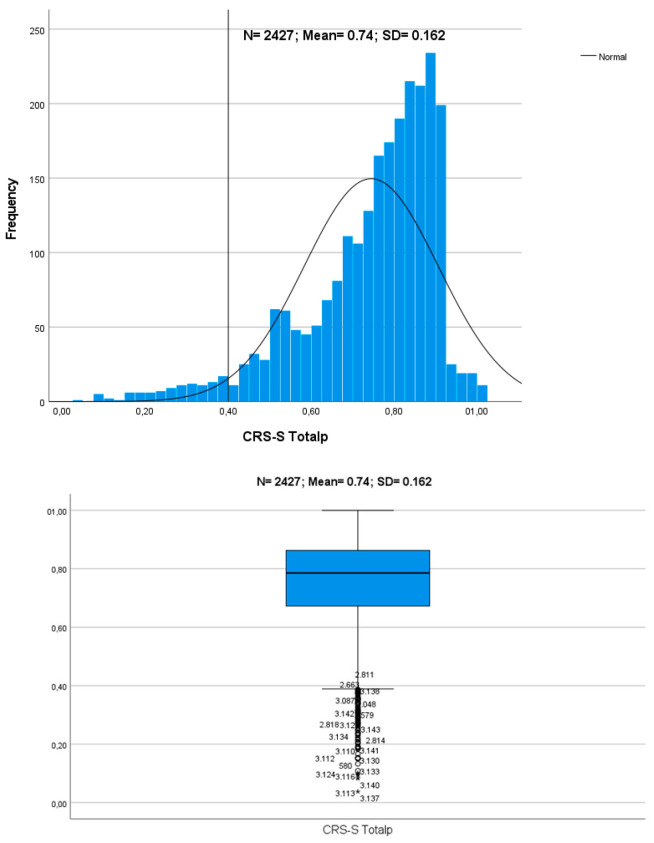

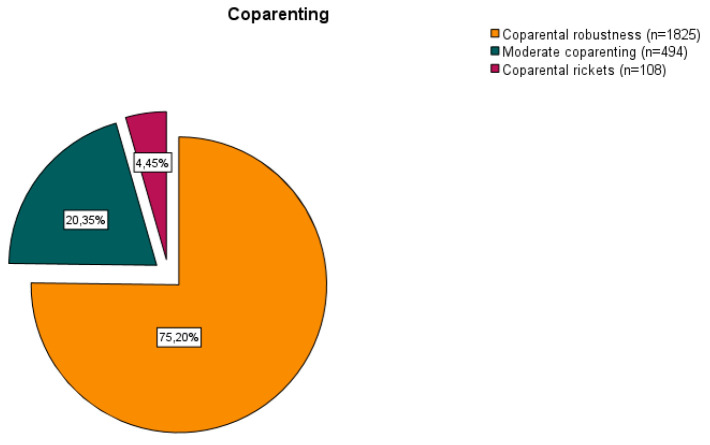
Distribution of the total weighted score for CRS-S in the group of participants: histogram, box plot, and percentage of participants in each of the three coparenting clusters derived from the result of the two-stage cluster analysis. *Note:* CRS-S = is the abbreviated way of naming CRS-S_Eg-S&D_. On the left chart, a dividing line is placed at 0.40. This value is the observed (approximate) value of the CRS-S Totalp distribution, below which are found the outliers and the extreme cases. The outliers (represented by circles) and extreme cases (represented by asterisks are visualized in the box plot graph in the center of Figure 1.

**Figure 2:**
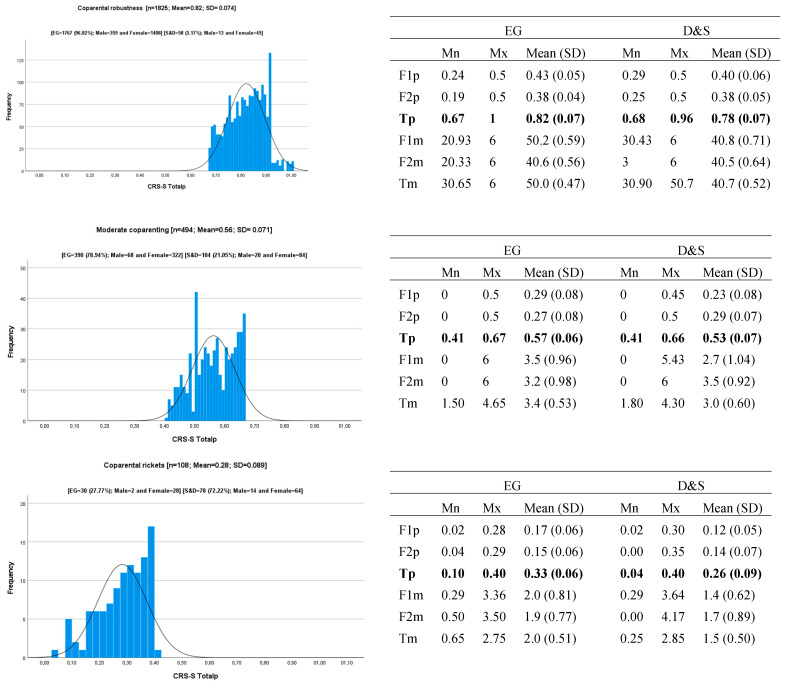
**Left panel**: For each of the three coparenting clusters, the distribution of the total weighted CRS-S score and description of demographic characteristics, sex, and marital status are shown. **Right panel**: For each of the three coparenting clusters, descriptive statistics of the two CRS-S dimensions and the total score (average and weighted scores) are shown. *Note*: In the tables, F1p, F2p, Tp, F1m, F2m and Tm = F1 CRS-Sp, F2 CRS-Sp, CRS-S Totalp, F1 CRS-Sm, F2 CRS-Sm, and CRS-S Totalm, respectively. The value of CRS-S Totalp is highlighted in bold because the distribution is represented in the graphs located in the middle part of this large table. For the rest, see Figure 1.

The result of the two-stage cluster analysis is conclusive. The classification quality reaches a value of 0.7, which in the terminology of Kaufman & Rousseeuw [87] is good. Therefore, it can be concluded that the data reasonably or strongly evidence the structure of the clusters. The *Coparental Vitality* measure calculated using the total weighted measure of CRS-S_Eg-S&D_ allows the sample of participants to be divided into three differentiated clusters that we have called *Coparental Robustness* or *Robust Coparenting*, *Moderate Coparenting*, and *Coparenting Rickets* or *Inadequate Coparenting*. MANOVA revealed that the effect size of the variable that defines the clusters is very big [cluster: Λ = 0.209; F = 1438.64 (*df*_1_; *df*_2_ = 4; 4846); *p* = 0.001; η^2^ = 0.543], and both dimensions were statistically significant with η^2^ = 0.656 (F = 2306.41) and η^2^ = 0.534 (F = 1391.14), respectively, for F1 CRS-Sp and F2 CRS-Sp (both df1 and df2 were 2 and 2424, and *p* < 0.001). Figure 1 and Figure 2 show the graphic representation of the distribution of CRS-S Totalp in each cluster and the descriptive statistics of each dimension (F1 CRS-S and F2 CRS-S, and Total CRS-S), referring to its weighted value and average value.

**Figure 3:**
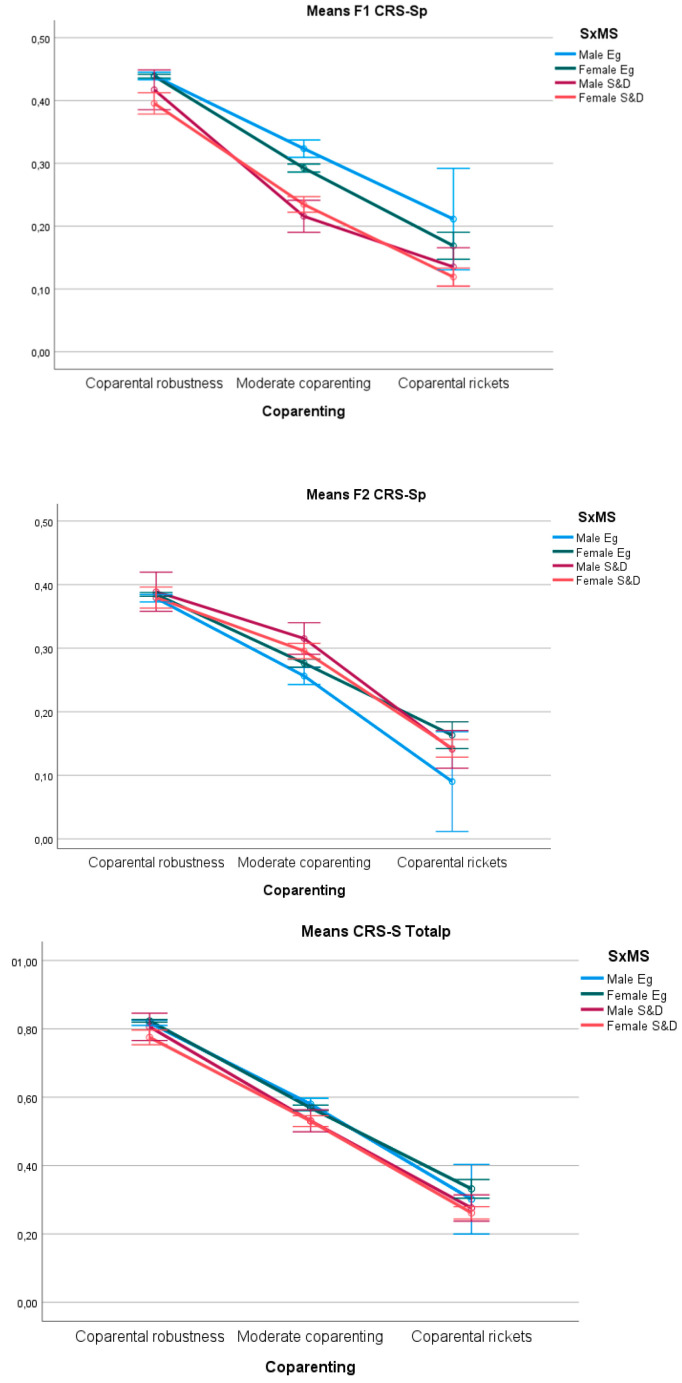
In order, representations of the scores F1 CSR-Sp, F2 CSRp, and CSR Totalp for the three profiles of coparental vitality in each subsample that is defined by the crossing of levels of the variables sex and marital status (SxMS). *Note:* See Figure 1 and Figure 2.

Figure 3 shows the representation of the F1 CSR-Sp, F2 CSRp, and CSR Totalp scores for the three *Coparental Vitality* profiles in each subsample defined by the crossing of levels of the variables sex and marital status (SxMS).

**Figure 4:**
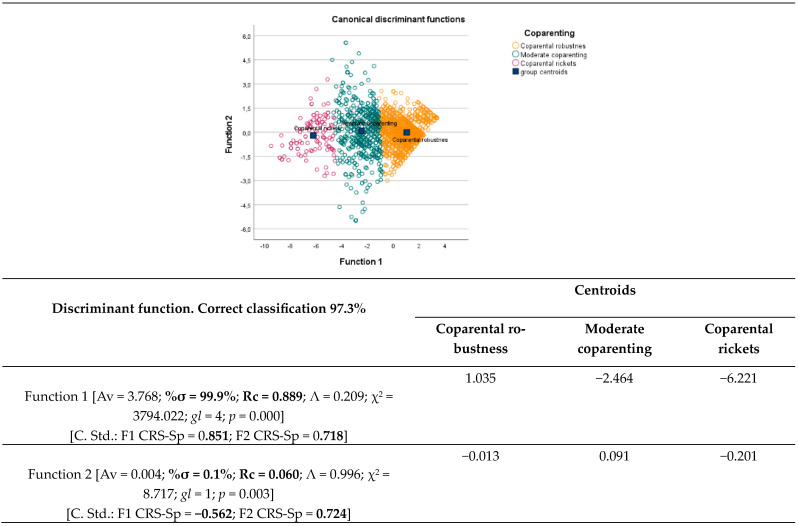
The upper part shows the graph of the distribution of the groups centroids of coparental vitality in the solution of the discriminant analysis carried out on the total sample. The bottom part shows a summary of the discriminant analysis (grouping variable = the three levels of coparental vitality derived from the two-stage cluster analysis; independent variables = F1 CRS-Sp and F2 CRS-Sp). *Note*: In the discriminant analysis, Av = eigenvalue; %σ = percentage of explained variance; Rc = canonical correlation; Λ = Wilks’ Lambda test statistic; *df* = degrees of freedom; and C. Std. = standardized coefficients of the relevant variables in the discrimination of the 3 groups, *Coparental robustness*, *Moderate coparenting*, and *Coparental rickets*. For the rest, see Figure 1 and Figure 2.

To evaluate the importance or strength of each of the two dimensions in the discrimination of the three levels of *Coparental Vitality*, a discriminant analysis was carried out [the grouping variable was Coparenting, with three levels reached by the two-stage cluster, and the independent variables were F1 CRS-Sp and F2 CRS-Sp]. The discriminant analysis (see Figure 4) revealed that two discriminant functions contribute to the differentiation of the three degrees of *Coparental Vitality*, with the contribution of the first (%σ = 99.9%; Rc = 0.889) being much higher than the second. In the first function, both variables, F1 SRC-Sp and F2 SRC-Sp, to a greater extent F1 SRC-Sp, contribute significantly to establishing a strong differentiation between the three levels of coparental vitality (see centroids in Figure 4). The second function, whose contribution is statistically significant but residual in magnitude (%σ = 0.1%; Rc = 0.060), is very revealing for two reasons. One reason is because it only contributes to differentiating *Coparental Rickets* from the other two levels of coparenting (see the sizes of the centroids), and the other reason is because the variable F2 SRC-Sp exerts a similar influence to that exerted in Function 1, but F1 CSR-Sp shows a negative value, which it could be interpreted as the substantial weakening of the measurement of the F1 SRC-Sp dimension that contributes the most to reach the state of *Rickets Coparenting* or *Inadequate Coparenting*.

## 4. Discussion and Conclusions

“The advantages of this measure will also facilitate the assessment of the domains of coparenting in clinical practice, allowing intervention to capitalize on areas of strength and focus on improving areas of difficulty” [26] (p. 12). Due to the great usefulness of the coparenting measure, and because the adaptation of the Coparenting Relationship Scale for the Spanish population carried out by Plá [37] has severe limitations, as highlighted in the Introduction, an instrumental investigation was carried out to adapt and validate the CRS to the Spanish population of engaged parents (Eg) and separated or divorced parents (S&D), evaluating the strength of the total measure of coparenting to classify the sample participants into different categories.

The research design was carefully planned, and extreme precautions were taken throughout the research process to guarantee the results’ replicability. An attempt has been made to avoid biases from the sample, the procedure, the data collection, and the bias caused by the analysis method. We think it is important to elaborate on the former in this Discussion section in three points.

First, it was verified through CFA and sCFA that the model found by Feinberg et al. [26] consisting of 30 items and six factors (Model CRS^6S^) does not fit satisfactorily to the Spanish population of Eg and S&D parents. A detailed study was then initiated to determine its dimensional structure. In the process, special attention was paid to two aspects: one that selected from the set of items those that had the greatest strength to capture the variability in the coparenting measure between all parents and that was valid for both sexes and for all civil marital statuses in which coparenting can take place, and the other that prevented the bias induced by the method or the sample from being responsible for the result found. Technically, taking care of both aspects will ensure the replicability of the result, and to achieve this, the dimensionality study was carried out in five steps. Initially, the sample was divided into two to conduct the cross-validation analysis, ensuring that sex and marital status were correctly balanced. Second, based on the descriptive statistics, all inappropriate items to describe the sample were eliminated. Third, consecutive EFAs were performed until a good model fit was achieved. The best-fitting model was called M1. M1 was then compared with other possible models (unidimensional and second-order factor), and the strength of the latent construct and its replicability were examined. Fourth, having found that M1 was a satisfactory model, it was tested using AFC and adjusted again, resulting in Model M2. Fifth, the factorial invariance of the M2 model was examined, and it was found that the M2 model has strong configurational, metric, scalar, and strict invariance for males and females and engaged parents and separated or divorced parents.

Second, we consider it necessary to study the dimensionality of the CRS in this way in the process of adaptation and validation of the sample of Spanish parents for two fundamental reasons. One is because the dimensional study by Feinberg et al. [26] was carried out in a sample with very particular characteristics that do not represent the set of parents in the population, heterosexual couples who, at the time of recruitment, were expecting their first child, a limitation that the authors highlighted, and based on this, they urged researchers to examine this structure in other samples. We must add that the sample size of the number of items was tiny (ratio 6.40:1). The second reason is despite the adaptation of the CRS that has been performed on multiple occasions, as stated in the Introduction, only three of them, Costa et al. [26], Dumitriu et al. [36], and Favez et al. [35] (excluding the one carried out by Plá, for the reasons previously indicated), have been performed for the entire population, men and women, Eg and D&S. We consider this aspect fundamental, given that otherwise the adaptation could be extended *ad infinitum* to all imaginable sample singularities. Therefore, we believe that the three cited investigations are the reference to contrast the results of this investigation.

Third, experts in the method have shown that a good model fit does not prove that the model is theoretically sound [64,80], that larger samples produce more precise solutions [98,99], that replication (perform the analysis using cross-validation and by evaluating the difference between factor loadings) avoids overfitting of the models and adds value to the result of the factor analysis [38,96], and that the EFA should always be a prior step to the CFA [42,100].

In this research, the proportion of Eg parents is much higher than that of S&D parents, and in each marital status, women are represented in a greater proportion than men. This also happens in the three reference investigations. The percentage of women is 70%, 63.40% and 56.9%, respectively, in Costa et al. [34], Dumitriu et al. [36], and Favez et al. [35], and the percentage of Eg is 80% in Costa et al. [34] and 69% in Dumitriu et al. [36] (Favez et al. [35] do not indicate this detail). However, this research distances itself from the three reference investigations in critical methodological aspects, which we summarize in two points.

The first is that the ratio between the sample size and the number of items is very large (41.3:1), being far removed from the research carried out by Costa et al. [34], Favez et al. [35] and Dumitriu et al. [36], which was 19.20:1, 11.4:1 and 14.4:1, respectively (the same occurs in research with parents of samples with unique characteristics, as shown in Section S4 of the Supplementary Materials).

The second is that none of the three conducts the analysis through cross-validation, and the value given to the descriptive analysis of the items to examine their suitability is not part of the scale (it is only performed to decide on the estimation method). None of them previously carried out an EFT, and in all three, the model of Feinberg et al. [26] of seven factors was tested directly using CFA.

Favez et al. [35], despite observing that items 13, 16, 21, and 22 were strongly skewed and had very high kurtosis, despite watching that they had a mean value very close to the lowest response value for the item, specifically 0.57, 0.51, 0.63 and 0.75 in the total sample, respectively, and that their SD was less than 1, they decided not to dispense with any item and use robust tests.

Costa et al. [34] and Dumitriu et al. [36] considered reducing the number of items on the scale as appropriate. Costa et al. [34] eliminated four items (items 13, 28, 5, and 20), and Dumitriu et al. [36] eliminated seven items (items 6, 7, 8, 28, 29, 5, and 20). Both were performed based on the factor loading or R^2^ observed in the CFA solution. Despite the eliminated items (only Costa et al. [34] decided to eliminate the Division of Labor factor), neither of the two investigations considered that the dimensionality was different due to removing items. In this research, ten items were eliminated, including the four eliminated by Costa et al. [34] and four (of seven) eliminated by Dumitriu et al. [36]. The Supplementary Materials (Section S4) describes the items destroyed in the investigations with singular samples.

Furthermore, Favez et al. [35] and Dumitriu et al. [36] conclude that the 7-factor Feinberg model fits their data, and Costa et al. [34] conclude that the model fits with six factors. However, in the three cases, the model is fitted tangentially (Costa et al. [34]: [χ^2^/*gl* = 4.69; CFI = 0 0.90; GFI = 0.85, RMSEA = 0.07], Dumitriu et al. [36] [χ^2^/*gl* = 3.90, RMSEA = 0 0.076] and Favez et al. [35] [χ^2^/*gl* = 2.94; CFI = 0.863; RMSEA = 0.07; SRMR = 0.078], and although experts emphasize that a well-fitted model does not indicate that the model is valid, none of these three investigations considered testing a different model. Feinberg et al. [26], Costa et al. [34], Favez et al. [35], and Dumitriu et al. [36], and the investigations carried out with singular samples too (see Section S4 in the Supplementary Materials) found a very high correlation between some factors, and that is a sufficient reason to think that another model perhaps fits better to the observed data.

Thus, it is concluded that the Model with 20 items sized in two factors is the simplest and best adjusted model for the sample of Spanish parents; it is invariant according to sex and marital status, and the measure derived from each of its factors is reliable and valid. The new questionnaire for Spanish parents is called CRS-S_Eg-S&D_. The resulting factors were defined as *Positive Coparenting* (F1, fourteen items) and *Negative Perception of Coparenting* (F2, six items), and their joint evaluation could indicate what we call *Coparental Vitality*.

The first factor has been called *Positive Coparenting* because it brings together items that refer to a parent’s cognition, feelings, and behaviors about the type of parental relationship they maintain with the other parent and the behaviors of the other that are necessary or facilitating for the exercise of positive coparenting. The higher the score, the greater the perception of harmony of the person responding regarding the exercise of co-parenting they are carrying out, and the more positive the perception they have of the behaviors and attitudes of the other parent as a father. The 14 items that make up F1, numbered with the number of the original scale of Feinberg et al. [26], are as follows:

2—My relationship with my partner is stronger now than before we had a child.

3—My partner asks my opinion on issues related to parenting.

6—My partner and I have the same goals for our child.

10—My partner tells me I am doing a good job or otherwise lets me know I am being a good parent.

14—My partner is sensitive to our child’s feelings and needs.

17—I feel close to my partner when I see him or her play with our child.

18—My partner has a lot of patience with our child.

19—We often discuss the best way to meet our child’s needs.

23—My partner is willing to make personal sacrifices to help take care of our child.

24—We are growing and maturing together through experiences as parents.

25—My partner appreciates how hard I work at being a good parent.

26—When I’m at my wits end as a parent, my partner gives me extra support I need.

27—My partner makes me feel like I’m the best possible parent for our child.

30—Parenting has given us a focus for the future.

The second factor has been called *Negative Perception of Coparenting* because the items that compose it establish discrepancies in parenting and parental behaviors that are incompatible with adequate coparenting. The higher the score on this factor, the greater the negative perception about the coparenting exercise they are performing.

The six items that make up F2, listed with the number of the original scale of Feinberg et al. [26], are as follows:

8—It is easier and more fun to play with the child alone than it is when my partner is present too.

9—My partner and I have different ideas about how to raise our child.

11—My partner and I have different ideas regarding our child’s eating, sleeping, and other routines.

12—My partner sometimes makes jokes or sarcastic comments about the way I am as a parent.

15—My partner and I have different standards for our child’s behavior.

29—My partner doesn’t like to be bothered by our child.

Factor 1 has items corresponding to four of the six factors contained in CRS^6S^ [*Coparenting Closeness*—items 2, 17, 24, and 30; *Endorse Partner Parenting*—items 14, 18, and 23; *Coparenting Support*—items 3, 10, 19, 25, 26, and 27, and *Coparenting Agreement*—item 6]. Factor 2 has items corresponding to three of the six factors contained in CRS^6S^ [*Coparenting Agreement*—items 9, 11, and 15; *Endorse Partner Parenting*—item 29, and *Coparenting Undermining*—items 8 and 12]. The only factor not represented is the *Division of Labor*, which also disappeared in the CRS adaptation process carried out by Costa et al. [34] (see Section 4 of the Supplementary Materials to see in which other investigations this factor disappeared too). Therefore, given that the two factors contain the items of five scales in Feinberg et al. [26], we consider that the theoretical model of Feinberg et al. [26] is correct. Still, the dimensional structure differs from the one they found.

On the other hand, Feinberg et al. [26] found that the *Exposure to Conflict* factor was positively related to the *Coparenting Undermining* factor (0.40 and 0.60 in the subsamples of fathers and mothers, respectively). They also found it was negatively associated with the *Coparenting Closeness* factor (−0.46 and −0.26 in the subsamples of fathers and mothers, respectively). The fact that the items that make up the *Coparenting Undermining* factor in Feinberg et al. [26] are part of Factor 2 and that the items that make up the *Coparenting Closeness* factor in Feinberg et al. [26] are part of Factor 1 supports, a priori, the argument presented in Section 2.5 *Data analysis* to avoid introducing the *Exposure to Conflict* factor was correct. However, this aspect must be studied in depth, as indicated below. Finally, research on the *Division of Labor* scale should be conducted to determine whether it truly adds value to the construct.

The *Coparental Vitality* measure calculated using the total weighted measure of CRS-S_Eg-S&D_ allows the sample of participants to be divided into three differentiated clusters that we have called *Coparental Robustness* (or *Robust Coparenting*), *Moderate Coparenting*, and *Coparenting Rickets* (or *Inadequate Coparenting*). Since this measure gives equal weight to both factors, it will allow the coparenting status of the parents to be evaluated more effectively.

Thus, considering that coparenting is a mediating factor between the couple’s relationship and the parents’ ability to adapt to their new roles and responsibilities [9], the result of the study presented here should be considered a starting point that requires future research on at least the following questions: (1) Study the differences between Eg and S&D parents in *Positive Coparenting* and *Negative Perception of Coparenting*, and examine if sex is a moderating variable of the differences (the proven factorial invariance allows this analysis to be carried out with guarantee). It is necessary to know if the total score resulting from the sum of the score achieved in the two factors (using the weighted measure) is capable of faithfully capturing the state of *Coparental Vitality* that a person describes and experiences, or on the contrary, the position reached should be taken into account in each of the two factors (the result of the discriminant analysis supports this last aspect more than the first). The analysis of the added value of the *Exposure to Conflict* scale must accompany the analysis described in this first point. As was made clear at the beginning of Section 2.5 *Data analysis*, only S&D people responded to the five items of this scale. It is necessary to examine whether the *Coparental Vitality* of S&D people can be expressed with the same depth with these items and without them. This is expected to be the case, among other things, due to the distribution of the factors found by Feinberg et al. [26] in the two factors found in this research, as described before. (2) Examine to what extent different variables (e.g., sexual orientation, culture, years divorced, number of children, religion or training received for parenting, being in child–parent reunification processes, and being adoptive parents) exert an added moderating effect on the previous differences [10,11,18]. (3) Evaluate the practical validity of the measure and its predictive validity to determine its usefulness as a diagnostic or assessment measure in different conditions (separation, divorce, adoption, child–parent reunification, the effectiveness of intervention programs for the exercise of positive coparenting with independence of the particularity of the family, such as parenting coordination, etc.) [1,2,26,30,101,102,103,104,105,106]. (4) Delve into the possibility of identifying a cut-off point based on which to determine when a coparenting relationship enters a state of risk [2,29]. (5) Determine how the coparenting relationship changes as the age of the children changes and with the passage of time [107,108]. (6) Determine how coparenting is different depending on the children’s problems (gifted children, autistic children, etc., in relation to children who do not have serious physical, cognitive, mental, or behavioral issues) [5,102,105].

Despite the control carried out throughout the research process, avoiding limitations has not been possible. The most notable is the asymmetry of participation based on sex. Although all parents, fathers, and mothers had the opportunity to participate in the study, self-selection could have occurred, affecting the results since this is a voluntary behavior.

However, based on the strength of the results found, despite the limitations, it can be concluded that the CRS-S_Eg-S&D_ questionnaire is reliable and valid for the study of coparenting in Spanish parents with children aged between 2 and 12 years.

## Figures and Tables

**Table 1:** Descriptive statistics of the items of *The Coparenting Relationship Scale* [26] that make up the questionnaire adapted to the Spanish population of engaged parents and separated or divorced parents, CRS-S_Eg-S&D_, and factor loadings of Model M1 in EFA, of Model M2 in CFA, and of the model invariant (Model M2, in CFA) to sex and to current marital status.

								Evaluation and Adjustment of CRS-S_Eg-S&D_ Dimensionality						Evaluation of Factorial Invariance of CRS-S_Eg-S&D._^V^ CFA M2, K = 20			
		Descriptive Statistics, IH_C_, Alpha						^C^ EFA M1; K = 21			^V^ EFA M1; K = 21	^V^ CFA M2; K = 20		^B^ Factor Loading by ^1^ Sex and ^2^ Marital Status			
Factors in M1 and M2	Items ^A^	M	SD	Skw	Kur	HI_C_	^I^ Alpha	F.Load.	Uniqu.	Com.	F.Load.	F.Load.	R^2^	Male	Female	Eg	S&D
	2	4.45	1.74	−0.91	−0.42	0.59	0.932	0.667	0.565	0.647	0.697	0.621	0.386	0.596	0.629	0.562	0.595
^D^ F1 (k = 14)	3	4.87	1.60	−1.34	0.60	0.69	0.931	0.657	0.470	0.834	0.732	0.769	0.591	0.756	0.770	0.708	0.785
	6	5.11	1.35	−1.60	1.76	0.69	0.931	0.628	0.471	0.789	0.680	0.739	0.546	0.629	0.758	0.664	0.763
	10	4.49	1.70	−0.85	−0.54	0.58	0.932	0.711	0.536	0.779	0.661	0.671	0.450	0.670	0.671	0.656	0.667
	14	4.91	1.56	−1.33	0.58	0.52	0.934	0.546	0.687	0.633	0.614	0.614	0.377	0.501	0.633	0.533	0.787
	17	5.03	1.55	−1.57	1.29	0.63	0.931	0.694	0.508	0.774	0.715	0.716	0.512	0.639	0.737	0.596	0.753
	18	4.39	1.60	−0.64	−0.79	0.61	0.932	0.656	0.553	0.750	0.664	0.714	0.510	0.634	0.729	0.659	0.811
	19	4.61	1.57	−1.00	−0.07	0.69	0.930	0.744	0.414	0.810	0.793	0.694	0.631	0.774	0.798	0.747	0.834
	23	4.72	1.69	−1.11	−0.09	0.66	0.931	0.713	0.481	0.834	0.723	0.728	0.530	0.642	0.743	0.641	0.822
	24	4.96	1.53	−1.47	1.18	0.75	0.929	0.843	0.297	0.911	0.877	0.847	0.717	0.855	0.845	0.790	0.852
	25	4.88	1.54	−1.32	0.65	0.75	0.929	0.894	0.248	0.897	0.862	0.850	0.722	0.884	0.844	0.803	0.895
	26	4.75	1.66	−1.16	0.10	0.78	0.929	0.869	0.251	0.955	0.870	0.891	0.794	0.901	0.889	0.856	0.899
	27	4.66	1.68	−1.10	−0.07	0.72	0.929	0.826	0.330	0.929	0.818	0.844	0.712	0.877	0.839	0.831	0.817
	30	4.44	1.78	−0.87	−0.56	0.55	0.933	0.652	0.603	0.690	0.687	0.632	0.389	0.602	0.632	0.573	0.536
	^i^ 8	4.27	1.46	−1.59	1.69	0.54	0.934	0.584	0.605	0.659	0.617	0.730	0.532	0.861	0.703	0.636	0.765
	^i^ 9	4.05	1.46	−1.41	1.34	0.64	0.933	0.774	0.356	0.799	0.610	0.764	0.583	0.695	0.755	0.723	0.905
	^i^ 11	4.13	1.44	−1.46	1.48	0.51	0.935	0.735	0.506	0.748	0.726	0.649	0.421	0.608	0.659	0.602	0.717
^D^ F2 (k = 6)	^i^ 12 ^D^	4.49	1.20	−2.06	4.48	0.52	0.939	0.664	0.566	0.693	0.688	0.575	0.331	0.533	0.579	0.585	0.609
	^i^ 15	3.92	1.48	−1.21	0.70	0.52	0.934	0.637	0.575	0.592	0.654	0.627	0.393	0.564	0.634	0.588	0.625
	^i^ 16	4.55	1.33	−2.25	4.62	0.44	0.936	0.617	0.665	0.715	0.581	-----	-----	-----	-----	-----	-----
	^i^ 29	4.38	1.25	−1.81	3.49	0.53	0.934	0.531	0.651	0.657	0.588	0.637	0.406	0.600	0.650	0.632	0.561

*Legend*. ^A^ = The number of the items corresponds to the numbering of the original scale [26] presented in the Supplementary Materials; ^i^ = inverse item; M, SD, Skw., Kur., HIc and ^I^ Alpha = mean, standard deviation, skewness, kurtosis, and corrected homogeneity index, respectively, and Cronbach’s alpha of the scale if the item is removed, respectively, found in the calibration sample; ^C^ = calibration sample (50% approx., n = 1239); ^V^ = validation sample (50% approx., N = 1188). ^1,2^ In the validation sample, male = 20.03% (n = 238), female = 79.96% (n = 950), and Eg = 90.067% (n = 1070), and S&D = 9.93% (n = 118); K = number of items in the tested model; k = number of items in the factor; F.Load. = factorial loadings; Uniqu. = uniqueness; Com. = comunalidad; ----- It was considered necessary to eliminate item 16 because R^2^ < 0.30 (R^2^ = 0.234) and standardized factorial loadings < 0.50 (F. Loading = 0.484); ^B^ = Standardized factorial loadings of M2 for male, female, Eg and S&D that were obtained in the configural invariance model are shown; ^D^ Crombach’s alpha [in M1 EFA (F1 = 0.943, and F2 = 0.838 and total test = 0.935] [in M2 CFA (F1 = 0.943 and F2 = 0.829 and total test = 0.936].

**Table 2:** Dimensionality models tested using EFA and CFA of the *The Coparenting Relationship Scale* [22] in the adaptation process to the Spanish population of engaged parents and separated or divorced parents, CRS-S_Eg-S&D_.

	MODEL	χ^2^ (*df*)	χ^2^/*df*	BIC/ECVI	CFI [TLI]	RMSEA [90%CI]	SRMR	^1^ S		
**^T^ CFA**	CRS^6S^ (K = 30)	2972.158 (390)	7.62	1312	0.968 [0.964]	0.052 [0.050–0.054]	0.071	0.195		
**^C^ EFA**	CRS^6S^ (K = 30)			−123.124	[0.881]	0.069 [0.066–0.071]		0.212		
**^C^ EFA**	M1 (K = 21)			**−0.536**	**[0.912]**	0.071 [0.067–0.074]		**0.999**		
**^V^ CFA**	M1 (K = 21)	203.218 (188) ^H^	1.08	0.328	0.999 [0.999]	0.008 [0–0.016]	0.035			
**^V^ CFA**	M2 (K = 20)	**176.660 (169) ^J^**	**1.04**	**0.226**	**1 [1]**	**0.006 [0–0.015]**	**0.034**			
**^V,3^ Invariance**	**M2 Sex**	**χ^2^ (*df*)**	**χ^2^/*df***		**CFI**	**RMSEA [90%CI]**	**SRMR**	**∆CFI**	**∆RMSEA**	**∆SRMR**
Conf. Invar.		227.651 (338)	0.673		1	0 [0–0]	0.040			
Metr. Invar.		282.733 (356)	0.794		1	0 [0–0]	0.044	0	0	0.004
Scal. Invar.		305.615 (374)	0.817		1	0 [0–0]	0.043	0	0	−0.001
Strict Invar.		317.394 (394)	0.805		1	0 [0–0]	0.044	0	0	0.001
**Invariance**	**M2 MS**									
Conf. Invar.		300.160 (338)	0.888		1	0 [0–0.005]	0.041			
Metr. Invar.		432.841 (356)	1.215		0.996	0.019 [0.012–0.025]	0.047	0.004	0.001	0.006
Scal. Invar.		505.343 (374)	1.351		0.994	0.024 [0.019–0.030]	0.047	0.002	0.005	0
Strict Invar.		594.265 (394)	1.508		0.991	0.029 [0.024–0.034]	0.050	0.003	0.005	0.003

*Legend*. χ^2^/*df* ratio [77]; BIC/ECVI = parsimony indices, BIC information criteria in EFA/expected cross-validation index in CFA; CFI [TLI] = comparative fit index [Tucker–Lewis index]; RMSEA = Root Mean Square Error of Approximation; SRMR = Standardized Root Mean Square of Residuals in CFA; S = Bentler’s simplicity index; ^1^ S was obtained through FACTOR (JASP does not provide the value); CRS^6S^ (K = 30), model found by Feinberg et al. [26], deleting the *Exposure to Conflict* scale. K = 30 items and 6 factors; ^T^ = total sample, N = 2427 participants; ^C^ = calibration sample (50% approx., n = 1239); ^V^ = validation sample (50% approx., N = 1188); M1 = best fitted model in the EFA (k = 21), where nine items were eliminated; M2 = the CFA of Model M1 fits well. However, it was considered necessary to eliminate item 16 (see the explanation in Table 1). ^3^ configural, metric, scalar, and strict invariance, respectively; ∆ comparison of the increment of the observed value in CFI, SRMR and RMSEA; MS = marital status (Eg and S&D); ^H^ *p* = 0.212; ^J^ *p* = 0.328. The best-fitting models of the EFA and CFA are highlighted in bold. For the rest, see Table 1.

**Table 3:** Correlation between the factors of CRS-S_Eg-S&D_, PAFAS, and CAPES and between the total score of the questionnaires. The correlations are shown in the set of men and women (without distinguishing between sexes) in the subsamples of engaged parents and separated or divorced parents.

		F1 CRS-S	F2 CRS-S	F11 PF	F12 PF	F13 PF	F21 PF	F22 PF	F1 CP	F2 CP	CRS-S-T	PF-T	CP-T
Eg	F1 CRS-S	1	0.404 **	0.150 **	0.182 **	0.267 **	0.320 **	0.601 **	−0.108 **	−0.253 **	0.860 **	0.510 **	−0.212 **
	F2 CRS-S		1	0.266 **	0.014	0.221 **	0.110 **	0.394 **	−0.156 **	−0.280 **	0.814 **	0.349 **	−0.217 **
	F11 PF			1	−0.038	0.183 **	0.052 *	0.151 **	−0.142 **	−0.370 **	0.244 **	0.508 **	−0.301 **
	F12 PF				1	0.248 **	0.170 **	0.171 **	−0.107 **	0.075 **	0.123 **	0.561 **	−0.047 *
	F13 PF					1	0.308 **	0.374 **	−0.197 **	−0.243 **	0.293 **	0.619 **	−0.067 **
	F21 PF						1	0.413 **	−0.099 **	−0.147 **	0.265 **	0.584 **	−0.159 **
	F22 PF							1	−0.168 **	−0.247 **	0.599 **	0.701 **	−0.265 **
	F1 CP								1	0.133 **	−0.154 **	−0.228 **	0.877 **
	F2 CP									1	−0.316 **	−0.303 **	0.593 **
	CRS-S-T										1	0.516 **	−0.283 **
	PF-T											1	−0.338 **
S&D	F1 CRS-S	1	0.497 **	−0.052	0.131 *	0.007	0.139 *	0.481 **	−0.113	−0.076	0.875 **	0.302 **	−0.137 **
	F2 CRS-S		1	0.049	−0.021	0.033	0.125	0.435 **	−0.077	−0.051	0.855 **	0.274 **	0.093 **
	F11 PF			1	−0.008	0.280 **	0.098	0.112	−0.102	−0.443 **	−0.004	0.517 **	−0.282 **
	F12 PF				1	0.294 **	0.197 **	0.073	−0.145*	−0.077	0.067	0.519 **	−0.136 *
	F13 PF					1	0.376 **	0.137 *	−0.261 **	−0.366 **	0.023	0.583 **	−0.372 **
	F21 PF						1	0.333 **	−0.177 **	−0.295 **	0.152 *	0.621 **	−0.292 **
	F22 PF							1	−0.176 *	−0.209 **	0.522 **	0.684 **	−0.241 **
	F1 CP								1	0.274 **	−0.110	−0.310 **	0.833 **
	F2 CP									1	−0.074	−0.456 **	0.694 **
	CRS-S-T										1	0.328 **	−0.134 **
	PF-T											1	−0.452 **

*Legend*. Eg and S&D = engaged parents and separated or divorced parents, respectively; CRS-S, PF and CP, is the abbreviated way of referring to CRS-S_Eg-S&D_, PAFAS, and CAPES in this table (for reasons of space); F1 and F2 in CRS-S are *Coparentalidad Positiva* and *Percepción Negativa de la Coparentalidad*, respectively, F11, F12, F13, F21, and F22 in PAFAS are *Family adjustment*, *Positive encouragement*, *Parent-child relationship*, *Parental adjustment*, and *Family adjustment*, respectively; F1 and F2 in CAPES are *Child’s competencies*, and *Behavioral and emotional problems*, respectively; CRS-S-T, PF-T and CP-T = total scores for the respective scales. All scores were transformed as indicated in the text (in PAFAS, each factor could achieve a maximum of 0.20, and in CRS-S_Eg-S&D_ and CAPES, each factor could achieve a maximum of 0.5, such that the total sum of each scale can reach a maximum value of 1); **^,^* = *p* < 0.01 and *p* ≤ 0.05, respectively Correlations greater than 0.50 have been highlighted in bold.

## Data Availability

The data presented in this study are available upon request from the corresponding authors. The data are not publicly available due to privacy reasons.

## Supplementary Materials

The following supporting information can be downloaded at https://www.mdpi.com/article/10.3390/children11050535/s1. Section S1: The Coparenting Relationship Scale (CSR) developed by Feinberg et al., (2012) [26], and translated to the Spanish adult population by Plá (2015) [37] (adapting the wording to our research to facilitate reading and responding). Section S2: The descriptive statistics of the items of six scales of the CRS questionnaire (Feinberg et al., 2012 [26]) in the responses of Spanish parents with children between the ages of 2 and 12 years (the Conflict Exposure scale is excluded). Due to the involvement of engaged parents (Eg) and separated or divorced parents (S&D) of both sexes, the results are presented independently for men and women of each marital status in the total sample (N = 2427) (Table S1), in the calibration subsample (n = 1239; 51.05%, approximately 50% of N) (Table S2), and in the validation subsample (n = 1188; 48.94%; approximately, 50% of the remaining sample of N, once the calibration sample has been extracted) (Table S3). The descriptive statistics are also presented for the total sample and the calibration and validation subsamples without differentiating between sexes or marital status (Table S4). Section S3: Table S5. Descriptive statistics of the items of six scales (the Exposure to Conflict scale is excluded) of the CRS questionnaire (Feinberg et al., 2012 [26]) in the calibration subsample (n = 1239), and pattern of factor loadings of the rotated matrix obtained through the EFA, requesting the same dimensionality of the six factors found by (Feinberg et al., 2012 [26]) (Table S5). The examination of the convergent and discriminant validity of the resulting dimensions in the adapted and validated questionnaire, CRS-SEg-S&D, examined through an empirical evaluation of the linear correlation with the factors that dimension the PAFAS questionnaires is also shown (Fariña et al., 2021 [57] and CAPES (Seijo et al., 2021 [59])) (Tables S6–S8). Section S4: This section shows the information that completes some paragraphs of the Discussion and Conclusions section of the article text. This added information is highlighted in red in for a better location. Section S5: Identification data of the participation of educational centers. Table S9. Exhaustive distribution of participation in schools is shown in the graph (left panel), and the distribution is ordered in participation intervals in a tabular way (right panel).
