# Supplementary material for: Adaptation of the Coparenting Relationship Scale Questionnaire to Spanish Parents with Offspring
Source: Children (Basel). 2024 Apr 30;11(5):535. doi: 10.3390/children11050535 (PMC11119224; doi:10.3390/children11050535)
Supplement: Supplementary file 1 [file children-11-00535-s001.zip › children-2918984-supplementary.pdf]

Adaptation of The Coparenting Relationship Scale Questionnaire to Spanish parents with offpring

Supplementary Material

Section S1. *The Coparenting Relationship Scale* (CSR) developed by Feinberg et al., (2012), and translated to the Spanish adult population by Plá (2015) (adapting the wording to our research to facilitate reading and responding).

Section S2. The descriptive statistics of the items of 6 scales of the CRS questionnaire (Feinberg et al., 2012) in the responses of Spanish parents with children between the ages of 2 and 12 (the Conflict Exposure scale is excluded). Due to involving engaged parents (Eg) and separated or divorced parents (S&D) of both sexes, the results are presented independently for men and women of each marital status in the total sample (N=2427) (Table S1), in the calibration subsample (n=1239; 51.05%. 50% approx. of N) (Table S2), in the validation subsample (n=1188; 48.94%. 50% approx., the remaining sample of N, once the calibration sample has been extracted) (Table S3). The descriptive statistics are also presented for the total sample and the calibration and validation subsamples without differentiating between sexes or marital status (Table S4).

Section S3. Table S5. Descriptive statistics of the items of 6 scales (the *Exposure to Conflict* scale is excluded) of the CRS questionnaire (Feinberg et al., 2012), in the calibration subsample (n=1239), and pattern of factor loadings of the rotated matrix obtained through the EFA, requesting the same dimensionality of 6 factors found by (Feinberg et al., 2012) (Table S5). Also is shown the examination of the convergent and discriminant validity of the resulting dimensions in the adapted and validated questionnaire, CRS-SEg-S&D, examined through the empirical evaluation of the linear correlation with the factors that dimension the PAFAS questionnaires (Fariña et al., 2021 and CAPES (Seijo et al.,2021) (Tables S6, S7 and S8)

Section S4. This section shows the information referred to in the third point of the article in section **4. Discussion and Conclusions.** This information is entered in the paragraphs that appear in the text of the article and are highlighted in red for a better location.

Section S5. Identification data of the participation of educational centers

Section S1. CRS. ***The Coparenting Relationship Scale*** (CSR) developed by **Feinberg et al., (2012),** and translated to the Spanish adult population by Plá (2015) (adapting the wording to our research to facilitate reading and responding). English [Spanish]

1. I believe my partner is a good parent [Creo que el otro progenitor es un/a buen/a padre/madre]
2. My relationship with my partner is stronger now than before we had a child [Mi relación con el otro progenitor es más fuerte ahora que antes de haber tenido un/a hijo/a]
3. My partner asks my opinion on issues related to parenting [El otro progenitor me pide mi opinión sobre cuestiones relacionados con la educación y cuidado de nuestro hijo/a]
4. My partner pays a great deal of attention to our child [El otro progenitor presta mucha atención a nuestro hijo/a]
5. My partner likes to play with our child and then leave dirty work to me [Al otro progenitor le gusta jugar con el niño pero deja el trabajo más desagradable para mí]
6. My partner and I have the same goals for our child [El otro progenitor y yo compartimos las mismas aspiraciones para nuestro hijo/a]
7. Partner still wants to do his or her own thing instead of being a responsable parent [El otro progenitor prefiere ir a lo suyo en vez de asumir su responsabilidad como madre/padre]
8. It is easier and more fun to play with the child alone than it is when my partner is present too [Es más fácil jugar con el niño cuando está solo que cuando está presente el otro progenitor]
9. My partner and I have different ideas about how to raise our child [El otro progenitor y yo tenemos distintas ideas acerca de cómo debemos criar a nuestro hijo/a.
10. My partner tells me I am doing a good job or otherwise lets me know I am being a good parent [El otro progenitor me dice que estoy haciendo una buena labor o me deja saber que soy un buen/a madre/padre.]
11. My partner and I have different ideas regarding our child’s eating, sleeping, and other routines [El otro progenitor y yo tenemos distintas ideas acerca de la alimentación, el sueño y otras rutinas de nuestro hijo/a]
12. My partner sometimes makes jokes or sarcastic comments about the way I am as a parent [El otro progenitor a menudo hace chistes o comentarios sarcásticos sobre mi manera de ser como madre/padre]
13. My partner does not trust my abilities as a parent [El otro progenitor no confía en mis habilidades como madre/padre]
14. My partner is sensitive to our child's feelings and needs [El otro progenitor es sensible a los sentimientos y necesidades de nuestro hijo/a]
15. My partner and I have different standards for our child’s behavior [El otro progenitor y yo tenemos distintos criterios en cuanto al comportamiento de nuestro hijo/a]
16. My partner tries to show that she or he is better than me at caring for our child [El otro progenitor intenta demostrar que sabe cuidar mejor de nuestro hijo/a que yo.
17. I feel close to my partner when I see him or her play with our child [Me siento cercano al otro progenitor cuando lo/a veo jugando con nuestro hijo/a]
18. My partner has a lot of patience with our child [El otro progenitor tiene mucha paciencia con nuestro hijo/a]
19. We often discuss the best way to meet our child’s needs [A menudo charlamos sobre la mejor manera de cubrir las necesidades de nuestro hijo/a]
20. My partner does not carry his or her fair share of the parenting work **[**El otro progenitor no asume su responsabilidad como madre/padre]
21. When all three of us are together, my partner sometimes competes with me for our child’s attention [Cuando estamos juntos los tres, algunas veces el otro progenitor compite conmigo para conseguir la atención de nuestro hijo/a]
22. My partner undermines my parenting [El otro progenitor cuestiona mi capacidad de ser madre/padre]
23. My partner is willing to make personal sacrifices to help take care of our child [El otro progenitor está dispuesto/a a hacer sacrificios personales para ayudar en el cuidado de nuestro hijo/a.]
24. We are growing and maturing together through experiences as parents [Estamos creciendo y madurando juntos a través de la experiencia de ser padres]
25. My partner appreciates how hard I work at being a good parent [El otro progenitor aprecia lo duro que trabajo para ser un buen/a madre/padre]

26. When I'm at my wits end as a parent, partner gives me extra support I need [Cuando no puedo más como madre/padre, el otro progenitor me aporta el apoyo que necesito]
27. My partner makes me feel like I'm best possible parent for our child [El otro progenitor me hace sentir que soy el/la mejor padre/madre para nuestro hijo/a.]
28. The stress of parenthood has caused my partner and me to grow apart [El estrés de ser padres nos ha distanciado como pareja]
29. My partner doesn't like to be bothered by our child [Al otro progenitor no le gusta que nuestro hijo/a le moleste]
30. Parenting has given us a focus for the future. [Ser padres nos ha centrado para conseguir un futuro.

ONLY in case of COUPLE BREAKUP, SEPARATION, OR DIVORCE, indicate the frequency of your behavior when you are both in the company of your child (e.g., in the same room, in the car,...) [SOLO en caso de RUPTURA DE PAREJA, SEPARACION O DIVORCIO, indica la frecuencia de vuestro comportamiento cuando ambos estáis en compañía de vuestro hijo/a (Ej., en la misma habitación, en el coche, …)]

31. Find yourself in a mildly tense or sarcastic interchange with your partner? [¿En los intercambios te encuentras ligeramente tenso o sarcástico con el otro progenitor?]
32. Argue with your partner about your child, in the child's presence? [¿Discutes con el otro progenitor acerca de vuestro hijo/a delante de él?]
33. Argue about your relationship or marital issues unrelated to your child, in the child's presence? [¿Discutes con el otro progenitor acerca de vuestra relación o sobre temas de la pareja que no están relacionados con vuestro hijo/a, delante de él?]
34. One or both of you say cruel or hurtful things to each other in front of the child? [¿Uno de vosotros o ambos os decís cosas crueles o que hieren al otro delante de vuestro hijo/a?]
35. Yell at each other within earshot of the child? ¿Os gritáis mutuamente delante de vuestro hijo/a?

Section S2

Table S1. Descriptive statistics of the 6-scale items (the *Exposure to Conflict* scale is excluded) from The Coparenting Relationship Scale (CRS) (Feinberg et al., 2012) **in the total sample set (N=2427)** in the parents' response referring to the youngest of their children (when the latter is 12 years old or younger). They are presented independently for men and women of each marital status, Eg and D&S.

		Eg Male (n=429; 17.76%)					Eg Female (n=1758; 72.43%)					D&S Male (n=47; 1.93%)					D&S Female (n=193; 7.95%)				
**Sb**	**Its[1]**	**M**	**SD**	**Skw**	**Kur**	**HIc**	**M**	**SD**	**Skw**	**Kur**	**HIc**	**M**	**SD**	**Skw**	**Kur**	**HIc**	**M**	**SD**	**Skw**	**Kur**	**HIc**
**F1**	6	5.42	.95	-1.89	3.47	.52	5.23	1.21	-1.74	2.53	.61	3.51	1.943	-.249	-.992	.57	3.35	1.848	.130	-1.340	.70
	[i]9	4.17	1.37	-1.54	2.07	.53	4.19	1.30	-1.63	2.37	.53	3.23	1.772	-.494	-.651	.64	2.73	1.837	-.172	-1.245	.67
	[i]11	4.16	1.35	-1.58	2.18	.45	4.25	1.31	-1.59	2.25	.44	3.11	1.948	-.230	-1.176	.67	3.11	1.884	-.445	-1.118	.54
	[i]15	4.06	1.36	-1.32	1.38	.48	4.06	1.32	-1.33	1.41	.43	3.34	1.845	-.353	-1.167	.47	2.92	1.782	-.350	-1.109	.58
**F2**	2	4.74	1.49	-1.19	.61	.37	4.68	1.57	-1.06	.07	.46	2.26	1.835	.531	-.853	.65	2.13	1.680	1.046	.064	.46
	17	5.13	1.42	-1.81	2.46	.42	5.30	1.26	-1.96	3.19	.53	2.81	1.974	.385	-1.051	.57	2.68	1.937	.550	-1.133	.65
	24	5.30	1.09	-1.92	4.21	.59	5.13	1.32	-1.56	1.61	.69	3.04	2.156	.065	-1.334	.74	2.54	1.955	.692	-.978	.67
	[i]28[B]	4.14	1.36	-1.36	1.34	.53	4.03	1.37	-1.27	1.04	.44	3.87	1.895	-.949	-.088	.06	3.12	2.204	-.330	-1.467	.22
	30	4.60	1.61	-1.00	-.12	.43	4.62	1.63	-.99	-.19	.48	2.98	2.202	.092	-1.488	.35	2.56	1.944	.603	-.978	.48
**F3**	3	5.26	1.14	-1.80	2.97	.52	5.05	1.44	-1.55	1.42	.62	3.11	2.139	.231	-1.476	.67	3.00	2.046	.287	-1.455	.76
	10	4.61	1.49	-.87	-.23	.47	4.59	1.65	-.89	-.46	.55	2.72	1.908	.297	-.957	.55	3.18	2.029	.094	-1.514	.68
	19	4.82	1.27	-1.07	.61	.53	4.79	1.43	-1.07	.17	.63	3.21	2.186	-.102	-1.426	.79	2.73	1.806	.436	-1.098	.73
	25	5.11	1.17	-1.38	1.33	.59	5.07	1.37	-1.47	1.22	.69	2.89	1.992	.153	-1.164	.78	2.87	2.015	.364	-1.354	.78
	26	5.17	1.22	-1.66	2.30	.61	4.91	1.46	-1.21	.30	.74	2.57	2.114	.490	-1.076	.81	2.33	1.899	.776	-.715	.75
	27	4.94	1.40	-1.33	.84	.62	4.79	1.55	-1.17	.21	.69	2.49	2.031	.642	-.862	.76	2.75	2.074	.447	-1.313	.74
**F4**	**1[A]**	5.70	.71	-3.61	16.90	.54	5.45	.98	-2.28	5.57	.69	3.89	1.879	-.599	-.741	.76	3.53	1.735	.133	-1.314	.72
	**4[A]**	5.70	.69	-2.96	10.47	.50	5.21	1.18	-1.71	2.58	.69	3.98	1.973	-.590	-.998	.68	3.23	1.777	.332	-1.159	.76
	**[i]7[A]**	4.81	1.03	-3.13	11.25	.52	4.49	1.26	-2.01	3.65	.56	3.57	2.008	-.762	-.816	.61	2.77	2.092	-.131	-1.495	.62
	14	5.26	1.32	-2.02	3.36	.28	4.96	1.52	-1.41	.88	.48	3.96	1.989	-.476	-1.122	.67	3.48	1.879	-.074	-1.334	.66
	18	4.80	1.36	-1.01	.12	.40	4.50	1.53	-.69	-.68	.60	2.87	1.884	.192	-1.159	.77	2.97	1.763	.322	-1.103	.67
	23	5.36	1.19	-2.24	4.57	.42	4.86	1.54	-1.21	.27	.59	3.15	2.157	.125	-1.491	.71	2.71	1.895	.605	-.987	.74
	[i]29	4.46	1.22	-2.07	4.77	.42	4.48	1.10	-1.90	4.51	.50	3.70	1.876	-.764	-.383	.51	3.52	1.753	-.713	-.548	.52
**F5**	[i]8	4.36	1.35	-1.57	2.04	.54	4.45	1.27	-1.88	3.12	.48	3.00	2.197	-.167	-1.528	.52	2.92	2.096	-.229	-1.463	.51
	[i]12	4.56	1.05	-2.02	4.96	.54	4.58	1.09	-2.26	5.89	.46	4.04	1.841	-1.091	.264	.53	3.83	1.754	-1.019	-.051	.52
	[i]13[B]	4.46	1.41	-2.08	3.64	.39	4.40	1.62	-1.97	2.56	.24	3.74	1.950	-.818	-.671	.65	3.59	1.961	-.777	-.791	.46
	[i]16[C]	4.57	1.25	-2.30	5.29	.49	4.68	1.15	-2.64	7.34	.37	3.66	2.003	-.813	-.742	.37	3.94	1.725	-1.250	.451	.39
	**[i]21[A]**	4.69	1.06	-2.55	7.83	.47	4.72	1.01	-2.63	8.21	.37	4.38	1.609	-1.578	1.876	.38	3.95	1.754	-1.163	.204	.37
	**[i]22[A]**	4.76	1.03	-2.90	10.04	.58	4.85	.95	-3.24	12.60	.39	4.11	1.879	-1.146	.190	.54	3.89	1.839	-1.084	-.002	.57
**F7**	**[i]5[A]**	3.79	1.63	-1.12	.16	.29	2.92	1.81	-.31	-1.26	.22	3.45	1.704	-.387	-.670	.13	2.33	1.946	.154	-1.421	.01
	[i]20[B]	4.59	1.44	-2.41	4.80	.32	4.47	1.51	-2.11	3.41	.30	3.83	1.785	-.905	-.154	.41	3.30	1.937	-.593	-1.006	.37

Legend. Its[1] = CRS scale items (Feinberg et al., 2012) exposed in Sections S1; Sb = Subscales found by (Feinberg et al., 2012) [F1= *Coparenting Agreement*; F2= *Coparenting Closeness*; F3= *Coparenting Support*; F4= *Endorsement of Partner Parenting*; F5= *Coparenting Undermining*; F7= *The Division of Labor*; Eg and S&D= Engaged parents and Separated or Divorced parents, respct; [i]= inverse items; Eg= Engaged parents; S&D= Separated or Divorced parents; M, SD, Skw., Kur. and HIc = Mean, standard deviation, skewness, kurtosis and corrected homogeneity index respect.; In Its[1], items are highlighted in bold (damaged statistics are highlighted in red) that for reasons of M, SD, Skw, Kur and IHc are not appropriate to form part of the dimensional structure of the scale ($M > 5.5$, SD<0.1 or very close to 1, Skw>3 y/o Cu>6 and CH_I <0.30). The letter indicates when they were eliminated [[A]= Before starting the EFA; [B]= in the development of the EFA, [C]= in the adjustment of the CFA] (see Table 1 in the Text).

Section 2

Table S2. Descriptive statistics of the 6-scale items (the *Exposure to Conflict* scale is excluded) from The Coparenting Relationship Scale (CRS) (Feinberg et al., 2012) **in the Calibration (N=1239)** in the parents' response referring to the youngest of their children (when the latter is 12 years old or younger). They are presented independently for men and women of each marital status, Eg and D&S.

		**Eg Male (n=214; 17.279%)**					**Eg Female (n=903; 72.88%)**					**D&S Male (n=24; 1.93%)**					**D&S Female (n=99; 7.90%)**				
**Sb**	**Its[1]**	**M**	**SD**	**Skw**	**Kur**	**HIc**	**M**	**SD**	**Skw**	**Kur**	**HIc**	**M**	**SD**	**Skw**	**Kur**	**HIc**	**M**	**SD**	**Skw**	**Kur**	**HIc**
**F1**	6	5.46	.87	-1.96	4.05	.52	5.27	1.17	-1.74	2.50	.59	3.29	1.99	.14	-1.28	.78	3.34	1.88	.15	-1.40	.64
	[i]9	4.14	1.39	-1.43	1.69	.58	4.22	1.29	-1.64	2.53	.56	3.00	1.89	-.59	-1.05	.83	2.51	1.86	-.04	-1.44	.62
	[i]11	4.16	1.41	-1.55	1.95	.50	4.27	1.31	-1.63	2.39	.43	2.83	2.01	-.13	-1.53	.84	3.10	1.88	-.41	-1.13	.49
	[i]15	3.98	1.49	-1.22	.76	.49	4.04	1.37	-1.34	1.28	.45	3.17	1.88	-.31	-1.32	.71	2.85	1.84	-.35	-1.22	.57
**F2**	2	4.81	1.42	-1.24	.84	.43	4.71	1.56	-1.11	.19	.45	2.17	1.95	.94	-.19	.68	1.90	1.43	1.24	.86	.44
	17	5.12	1.39	-1.75	2.32	.42	5.33	1.23	-2.01	3.39	.50	2.71	1.94	.64	-.76	.53	2.67	1.93	.59	-1.18	.64
	24	5.33	1.07	-1.99	4.64	.60	5.19	1.25	-1.62	1.96	.68	3.13	2.13	.03	-1.25	.73	2.42	1.86	.79	-.77	.57
	[i]28[B]	4.12	1.38	-1.40	1.34	.58	4.04	1.38	-1.29	.98	.45	3.96	1.65	-1.06	.89	.29	3.16	2.23	-.32	-1.52	.18
	30	4.67	1.58	-.98	-.25	.47	4.64	1.64	-1.02	-.14	.46	3.25	2.35	-.04	-1.72	.27	2.43	1.96	.75	-.89	.53
**F3**	3	5.24	1.14	-1.58	1.69	.50	5.05	1.44	-1.53	1.37	.59	3.21	2.13	.30	-1.57	.78	2.90	2.05	.38	-1.40	.77
	10	4.59	1.49	-.86	-.25	.42	4.66	1.61	-.98	-.27	.52	2.58	1.84	.54	-.66	.63	3.17	2.04	.16	-1.52	.72
	19	4.81	1.35	-1.22	.98	.52	4.82	1.40	-1.09	.22	.60	3.29	2.05	.00	-1.26	.87	2.56	1.75	.59	-1.02	.74
	25	5.10	1.15	-1.22	.51	.58	5.12	1.32	-1.55	1.52	.68	3.13	1.92	.17	-1.21	.79	2.62	1.95	.61	-1.11	.74
	26	5.21	1.18	-1.61	1.91	.56	4.98	1.43	-1.27	.41	.72	2.75	2.23	.45	-1.34	.81	2.16	1.77	.95	-.30	.68
	27	4.98	1.35	-1.38	.97	.57	4.86	1.52	-1.27	.51	.64	2.63	1.91	.75	-.61	.74	2.61	2.01	.61	-1.13	.75
**F4**	**1[A]**	5.68	.69	-3.20	14.03	.57	5.47	.95	-2.21	5.27	.70	3.96	1.90	-.64	-.70	.84	3.57	1.72	.09	-1.35	.69
	**4[A]**	5.71	.62	-2.57	7.22	.46	5.21	1.16	-1.61	2.23	.67	3.83	1.93	-.30	-1.39	.74	3.12	1.74	.41	-1.04	.69
	**[i]7[A]**	4.77	1.17	-3.04	9.56	.53	4.48	1.28	-1.93	3.31	.61	3.42	1.98	-.82	-.79	.67	2.59	2.03	-.01	-1.53	.62
	14	5.31	1.26	-2.17	4.16	.22	4.99	1.48	-1.42	.94	.45	3.83	1.95	-.17	-1.57	.67	3.52	1.85	-.06	-1.36	.58
	18	4.79	1.37	-1.03	.19	.39	4.49	1.53	-.63	-.81	.58	2.71	1.83	.52	-.84	.78	3.01	1.78	.31	-1.26	.60
	23	5.41	1.12	-2.39	5.58	.40	4.83	1.56	-1.19	.23	.58	2.92	2.06	.45	-1.33	.71	2.62	1.84	.70	-.87	.73
	[i]29	4.43	1.31	-2.07	4.40	.45	4.49	1.11	-1.95	4.64	.50	3.83	1.71	-.85	.18	.67	3.45	1.69	-.67	-.44	.54
**F5**	[i]8	4.27	1.45	-1.49	1.49	.48	4.45	1.26	-1.84	2.98	.49	2.92	1.95	-.14	-1.32	.51	2.95	2.08	-.29	-1.46	.41
	[i]12	4.52	1.10	-1.91	4.13	.61	4.59	1.07	-2.28	6.22	.47	3.87	1.98	-.94	-.32	.62	3.69	1.78	-.91	-.33	.50
	[i]13[B]	4.39	1.49	-1.93	2.74	.40	4.40	1.64	-1.95	2.43	.27	3.63	1.91	-.85	-.83	.75	3.44	1.97	-.66	-1.04	.42
	[i]16[C]	4.47	1.38	-2.04	3.68	.50	4.67	1.20	-2.62	6.94	.39	3.50	2.02	-.79	-.90	.45	3.79	1.80	-1.13	.08	.36
	**[i]21[A]**	4.62	1.20	-2.49	6.62	.47	4.72	1.03	-2.62	7.95	.37	4.08	1.82	-1.23	.39	.43	3.93	1.72	-1.17	.29	.30
	**[i]22[A]**	4.71	1.15	-2.81	8.58	.59	4.84	.98	-3.14	11.53	.43	3.67	1.99	-.93	-.45	.68	3.90	1.74	-1.18	.37	.56
**F7**	**[i]5[A]**	3.84	1.62	-1.17	.23	.32	2.91	1.78	-.25	-1.27	.26	3.08	1.50	-.32	-.93	-.11	2.08	1.92	.34	-1.38	-.09
	[i]20[B]	4.54	1.50	-2.34	4.30	.27	4.43	1.56	-2.00	2.83	.33	3.21	1.98	-.46	-1.00	.55	2.98	1.99	-.36	-1.41	.26

Legend. See Table S1

Section 2

Table S3. Descriptive statistics of the 6-scale items (the *Exposure to Conflict* scale is excluded) from The Coparenting Relationship Scale (CRS) (Feinberg et al., 2012) **in the Validation (N=1188)** in the parents' response referring to the youngest of their children (when the latter is 12 years old or younger). They are presented independently for men and women of each marital status, Eg and D&S.

		**Eg Male (n=215; 18.09%)**					**Eg Female (n=855; 71.96%)**					**D&S Male (n=23; 1.93%)**					**D&S Female (n=95; 7.99%)**				
**Sb**	**Its[1]**	**M**	**SD**	**Skw**	**Kur**	**HI$_C$**	**M**	**SD**	**Skw**	**Kur**	**HI$_C$**	**M**	**SD**	**Skw**	**Kur**	**HI$_C$**	**M**	**SD**	**Skw**	**Kur**	**HI$_C$**
**F1**	6	5.37	1.01	-1.80	2.93	.53	5.19	1.26	-1.73	2.48	.62	3.74	1.91	-.70	-.16	.29	3.36	1.83	.12	-1.28	.76
	[i]9	4.20	1.35	-1.67	2.57	.48	4.17	1.32	-1.62	2.23	.51	3.48	1.65	-.26	-.42	.38	2.96	1.80	-.31	-.97	.72
	[i]11	4.16	1.29	-1.61	2.51	.39	4.22	1.31	-1.55	2.13	.44	3.39	1.88	-.31	-.73	.45	3.13	1.90	-.49	-1.10	.59
	[i]15	4.14	1.22	-1.38	2.12	.47	4.09	1.26	-1.30	1.51	.41	3.52	1.83	-.43	-.99	.18	3.00	1.73	-.34	-1.00	.59
**F2**	2	4.67	1.56	-1.14	.40	.33	4.66	1.58	-1.01	-.05	.47	2.35	1.75	.03	-1.58	.62	2.37	1.88	.81	-.59	.47
	17	5.15	1.45	-1.87	2.66	.43	5.26	1.29	-1.92	3.00	.57	2.91	2.04	.16	-1.19	.62	2.68	1.95	.52	-1.09	.66
	24	5.27	1.11	-1.87	3.93	.59	5.07	1.39	-1.48	1.26	.71	2.96	2.23	.11	-1.43	.79	2.67	2.05	.60	-1.16	.76
	[i]28[B]	4.15	1.35	-1.32	1.40	.47	4.02	1.36	-1.26	1.13	.43	3.78	2.15	-.86	-.63	-.14	3.07	2.18	-.35	-1.43	.28
	30	4.53	1.63	-1.02	-.01	.40	4.61	1.62	-.97	-.23	.50	2.70	2.05	.17	-1.26	.52	2.69	1.92	.47	-.99	.44
**F3**	3	5.29	1.13	-2.03	4.44	.53	5.06	1.44	-1.56	1.48	.65	3.00	2.20	.20	-1.49	.56	3.11	2.05	.20	-1.50	.74
	10	4.62	1.50	-.90	-.18	.52	4.50	1.68	-.81	-.62	.57	2.87	2.01	.08	-1.06	.47	3.19	2.03	.02	-1.53	.66
	19	4.83	1.19	-.84	-.15	.55	4.76	1.45	-1.06	.12	.66	3.13	2.36	-.15	-1.66	.75	2.91	1.85	.29	-1.12	.71
	25	5.12	1.20	-1.53	2.09	.61	5.01	1.42	-1.39	.94	.71	2.65	2.08	.21	-1.17	.82	3.13	2.06	.13	-1.46	.81
	26	5.13	1.26	-1.70	2.59	.65	4.84	1.49	-1.15	.20	.77	2.39	2.02	.54	-.76	.84	2.49	2.02	.62	-1.02	.80
	27	4.89	1.45	-1.28	.74	.67	4.72	1.58	-1.08	-.05	.73	2.35	2.19	.65	-.99	.82	2.89	2.14	.29	-1.44	.73
**F4**	**1[A]**	5.72	.73	-4.00	19.80	.51	5.43	1.02	-2.33	5.72	.69	3.83	1.90	-.60	-.66	.68	3.49	1.76	.18	-1.28	.77
	**4[A]**	5.69	.75	-3.12	11.38	.55	5.22	1.21	-1.80	2.90	.71	4.13	2.05	-.92	-.43	.60	3.34	1.82	.25	-1.26	.82
	**[i]7[A]**	4.84	.88	-3.00	12.21	.51	4.51	1.24	-2.09	4.07	.51	3.74	2.07	-.79	-.75	.55	2.95	2.15	-.27	-1.44	.62
	14	5.20	1.37	-1.89	2.80	.35	4.93	1.57	-1.40	.80	.50	4.09	2.07	-.81	-.56	.69	3.44	1.92	-.08	-1.32	.75
	18	4.82	1.36	-1.00	.07	.40	4.51	1.54	-.76	-.54	.62	3.04	1.97	-.11	-1.23	.76	2.94	1.76	.34	-.93	.76
	23	5.32	1.27	-2.11	3.84	.45	4.89	1.52	-1.24	.31	.60	3.39	2.27	-.18	-1.51	.73	2.80	1.95	.52	-1.09	.75
	[i]29	4.48	1.11	-2.03	5.01	.39	4.47	1.08	-1.85	4.41	.51	3.57	2.06	-.68	-.73	.38	3.59	1.82	-.77	-.59	.50
**F5**	[i]8	4.45	1.24	-1.62	2.64	.61	4.44	1.29	-1.91	3.26	.46	3.09	2.47	-.23	-1.78	.55	2.89	2.12	-.17	-1.48	.61
	[i]12	4.60	.99	-2.15	6.13	.45	4.57	1.11	-2.24	5.59	.46	4.22	1.70	-1.34	1.60	.38	3.98	1.72	-1.16	.39	.54
	[i]13[B]	4.53	1.33	-2.28	4.92	.38	4.41	1.60	-1.99	2.74	.20	3.87	2.03	-.88	-.40	.55	3.75	1.95	-.93	-.44	.49
	[i]16[C]	4.66	1.10	-2.60	7.84	.49	4.68	1.11	-2.66	7.83	.34	3.83	2.01	-.91	-.43	.25	4.09	1.64	-1.40	1.02	.42
	**[i]21[A]**	4.76	.89	-2.32	7.65	.49	4.71	.99	-2.63	8.56	.38	4.70	1.33	-2.18	6.46	.28	3.98	1.79	-1.18	.19	.43
	**[i]22[A]**	4.81	.89	-2.82	10.97	.57	4.85	.92	-3.37	13.98	.34	4.57	1.67	-1.52	1.73	.33	3.87	1.95	-1.02	-.26	.58
**F7**	**[i]5[A]**	3.74	1.64	-1.07	.13	.27	2.93	1.83	-.36	-1.25	.18	3.83	1.85	-.71	-.22	.36	2.59	1.94	-.03	-1.37	.07
	[i]20[B]	4.63	1.38	-2.50	5.48	.38	4.52	1.45	-2.26	4.16	.27	4.48	1.31	-1.28	1.50	.19	3.63	1.828	-.867	-.296	.46

Legend. See Table S1

Section 2

Table S4. Descriptive statistics of the 6-scale items (the *Exposure to Conflict* scale is excluded) from The Coparenting Relationship Scale (CRS) (Feinberg et al., 2012) of the set of participants **that make up the total sample (N=2427) (without differentiating between sex or marital status) in the calibration and validation subsamples used in the development of the FA.**

		**Total sample (N=2427)**						**Calibration sample (n=1239; 51.05%)**						**Validation sample (n=1188; 48.94%)**				
**Sb**	**Its**[1]	**M**	**SD**	**Skw**	**Kur**	**HI_C**		**M**	**SD**	**Skw**	**Kur**	**HI_C**		**M**	**SD**	**Skw**	**Kur**	**HI_C**
**F1**	6	5.08	1.37	-1.57	1.68			5.11	1.35	-1.60	1.76	.69		5.05	1.40	-1.55	1.60	.69
	[i]9	4.06	1.43	-1.43	1.41			4.05	1.46	-1.41	1.34	.64		4.06	1.41	-1.45	1.49	.57
	[i]11	4.12	1.43	-1.44	1.51			4.13	1.44	-1.46	1.48	.51		4.11	1.41	-1.43	1.54	.50
	[i]15	3.96	1.42	-1.23	.91			3.92	1.48	-1.21	.70	.52		4.00	1.35	-1.22	1.11	.48
**F2**	2	4.44	1.74	-.89	-.43			4.45	1.74	-.91	-.42	.59		4.43	1.74	-.87	-.44	.55
	17	5.01	1.57	-1.56	1.28			5.03	1.55	-1.57	1.29	.63		4.99	1.59	-1.55	1.27	.66
	24	4.92	1.56	-1.41	.95			4.96	1.53	-1.47	1.18	.75		4.87	1.59	-1.35	.74	.77
	[i]28[B]	3.97	1.49	-1.23	.77			3.98	1.49	-1.24	.70	.43		3.96	1.48	-1.22	.85	.42
	30	4.42	1.77	-.86	-.54			4.44	1.78	-.87	-.56	.55		4.40	1.75	-.84	-.52	.54
**F3**	3	4.89	1.59	-1.38	.73			4.87	1.60	-1.34	.60	.69		4.90	1.59	-1.41	.87	.71
	10	4.44	1.72	-.81	-.61			4.49	1.70	-.85	-.54	.58		4.39	1.74	-.76	-.67	.60
	19	4.60	1.57	-.99	-.04			4.61	1.57	-1.00	-.07	.69		4.59	1.56	-.99	.01	.71
	25	4.86	1.56	-1.31	.64			4.88	1.54	-1.32	.65	.75		4.83	1.58	-1.29	.63	.76
	26	4.71	1.67	-1.13	.07			4.75	1.66	-1.16	.10	.78		4.66	1.68	-1.11	.04	.81
	27	4.61	1.71	-1.05	-.17			4.66	1.68	-1.10	-.07	.72		4.56	1.73	-1.00	-.26	.76
**F4**	**1[A]**	5.31	1.19	-2.04	3.68			5.33	1.16	-2.03	3.72	.76		5.30	1.23	-2.05	3.62	.75
	**4[A]**	5.12	1.34	-1.62	1.88			5.10	1.33	-1.56	1.67	.73		5.13	1.35	-1.69	2.10	.76
	**[i]7[A]**	4.39	1.42	-1.87	2.72			4.36	1.46	-1.78	2.33	.67		4.43	1.38	-1.96	3.20	.60
	14	4.88	1.59	-1.32	.55			4.91	1.56	-1.33	.58	.52		4.84	1.63	-1.31	.50	.57
	18	4.40	1.61	-.68	-.69			4.39	1.60	-.64	-.79	.61		4.41	1.61	-.73	-.58	.66
	23	4.75	1.67	-1.14	-.01			4.72	1.69	-1.11	-.09	.66		4.77	1.66	-1.17	.08	.67
	[i]29	4.38	1.23	-1.81	3.52			4.38	1.25	-1.81	3.49	.53		4.38	1.22	-1.81	3.57	.52
**F5**	[i]8	4.28	1.46	-1.61	1.80			4.27	1.46	-1.59	1.69	.54		4.29	1.47	-1.64	1.92	.57
	[i]12	4.51	1.19	-2.08	4.62			4.49	1.20	-2.06	4.48	.52		4.52	1.17	-2.11	4.81	.47
	[i]13[B]	4.33	1.64	-1.82	2.03			4.30	1.67	-1.76	1.73	.36		4.37	1.60	-1.88	2.39	.30
	[i]16[C]	4.58	1.27	-2.34	5.33			4.55	1.33	-2.25	4.62	.44		4.61	1.19	-2.43	6.18	.39
	**[i]21[A]**	4.64	1.13	-2.43	6.47			4.63	1.17	-2.39	6.01	.41		4.66	1.09	-2.46	7.00	.42
	**[i]22[A]**	4.74	1.12	-2.78	8.42			4.72	1.15	-2.71	7.76	.53		4.76	1.08	-2.86	9.22	.47
**F7**	**[i]5[A]**	3.04	1.83	-.39	-1.20			3.01	1.81	-.34	-1.25	.25		3.07	1.84	-.45	-1.15	.19
	[i]20[B]	4.39	1.58	-1.91	2.51			4.31	1.65	-1.76	1.82	.40		4.46	1.49	-2.09	3.43	.34

Legend. See Table S1.

Section 3

Table S5.- Descriptive statistics of the items of 6 scales (the *Exposure to Conflict* scale is excluded) of the CRS questionnaire (Feinberg et al., 2012), in the calibration subsample (n=1239), and pattern of factor loadings of the rotated matrix obtained through the EFA, requesting the same dimensionality of 6 factors found by (Feinberg et al., 2012).

		Descriptive statistics, IH$_C$						[3] Factor loadings of the items in the application of 6 factors on the CRS scale (Feinberg et al.,2012)					
**Sb**	**Its[1]**	**M**	**SD**	**Skw**	**Kur**	**HI$_C$**	**Íts[2]**	**F1**	**F2**	**F3**	**F4**	**F5**	**F6**
**F1**	6	5.11	1.35	-1.60	1.76	.69	6		.549				
	^{i}9	4.05	1.46	-1.41	1.34	.64	^{i}9				.840		
	^{i}11	4.13	1.44	-1.46	1.48	.51	^{i}11				.559		
	^{i}15	3.92	1.48	-1.21	.70	.52	^{i}15				.537		
**F2**	2	4.45	1.74	-.91	-.42	.59	2					.713	
	17	5.03	1.55	-1.57	1.29	.63	**17***		.351				
	24	4.96	1.53	-1.47	1.18	.75	**24***					.372	
	^{i}28^{B}	3.98	1.49	-1.24	.70	.43	**^{i}28***					.331	
	30	4.44	1.78	-.87	-.56	.55	30					.501	
**F3**	3	4.87	1.60	-1.34	.60	.69	3		.449				
	10	4.49	1.70	-.85	-.54	.58	10	.704					
	19	4.61	1.57	-1.00	-.07	.69	**19**$^{\wedge}$	.347	.361				
	25	4.88	1.54	-1.32	.65	.75	25	.743					
	26	4.75	1.66	-1.16	.10	.78	26	.588					
	27	4.66	1.68	-1.10	-.07	.72	27	.924					
**F4**	**1^{A}**	5.33	1.16	-2.03	3.72	.76	1		.600				
	**4^{A}**	5.10	1.33	-1.56	1.67	.73	4		.609				
	**^{i}7^{A}**	4.36	1.46	-1.78	2.33	.67	**^{i}7^***				.373		.377
	14	4.91	1.56	-1.33	.58	.52	14		.486				
	18	4.39	1.60	-.64	-.79	.61	18		.402				
	23	4.72	1.69	-1.11	-.09	.66	**23^***	.323	.317				.331
	^{i}29	4.38	1.25	-1.81	3.49	.53	**^{i}29^{-}**						
**F5**	^{i}8	4.27	1.46	-1.59	1.69	.54	**^{i}8^***			.340	.336		
	^{i}12	4.49	1.20	-2.06	4.48	.52	^{i}12			.593	.310		
	^{i}13^{B}	4.30	1.67	-1.76	1.73	.36	^{i}13			.622			
	^{i}16^{C}	4.55	1.33	-2.25	4.62	.44	^{i}16			.503			
	**^{i}21^{A}**	4.63	1.17	-2.39	6.01	.41	^{i}21			.769			
	**^{i}22^{A}**	4.72	1.15	-2.71	7.76	.53	^{i}22^{-}						
**F7**	**^{i}5^{A}**	3.01	1.81	-.34	-1.25	.25	**^{i}5***						.311
	^{i}20^{B}	4.31	1.65	-1.76	1.82	.40	^{i}20		.549				

Scree Plot

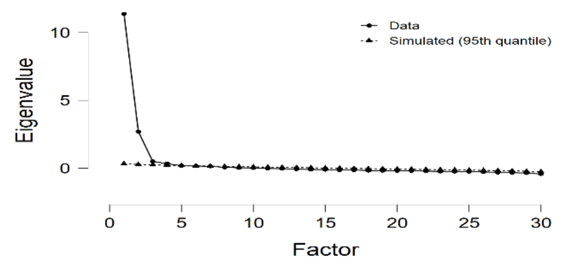

Factor correlations

	**%σ**	**F2**	**F3**	**F4**	**F5**	**F6**
**F1**	**.280**	**.720**	.239	.483	**.832**	.377
**F2**	**.111**		.245	.432	**.749**	.436
**F3**	.044			**.831**	.337	.370
**F4**	.043				**.511**	.484
**F5**	.034					.384
F6	.029					

Leyenda: In Its[2] highlight the items that in the initial 6-factor model request are: ^ = complex items (they share load in more than one factor and the difference between loads is <0.25; - = items that do not show load (loads factorial ≤ 0.30); * = items with factorial loads less than <0.40; [3]= Factor loadings less than 0.40 have been highlighted in red. If these factor loadings were not considered, factors F1 and F3 would have an item that they would share with another different factor. It can also be seen that F2 and F4 would lose 3 items each, F3 and F7 would lose 1 item each, and F5 would lose 2 items. If we stop to examine the correlation between the 6 requested factors, the factors F1, F2, and F5 have a very high correlation with each other, and also F3 and F4, making it possible to suspect that each is a linear combination of the others. This aspect, added to the descriptive study of the items presented in Tables S1-S4, leads us to establish that in the sample of Spanish parents some items are not valid to form the dimensionality of coparenting, to the extent that was found by Feinberg et al. (2012). The fit of the models tested in carrying out the EFA and CFA is in Tables 1 and 2 of the text. For the rest, see Table S1; The internal consistency examined using the standardized Cronbach's Alpha of the subscales Coparenting Agreement, Coparenting Closeness, Coparenting Support, Endorsement of Partner Parenting, Coparenting Undermining, and The Division of Labor was .764, .780, .916, .866, .803, .282, and the total scale .943

Section 3

Table S6. In the total sample. Empirical correlations between the factors of the CRS questionnaire adapted and validated for the population of Spanish parents, CRS-S$_{Eg\text{-}S\&D}$, and the factors of the PAFAS (5 factors) and CAPES (two factors) questionnaires. Correlations between the total scores of the scales are also shown. The results are presented segmented according to marital status, combining men and women in the estimation. Correlations greater than .50 stand out (Mukaka, 2012)

		F1 CRS-S	F2 CRS-S	F11 PF	F12 PF	F13 PF	F21 PF	F22 PF	F1 CP	F2 CP	CRS-S-T	PF-T	CP-T
Eg	F1 CRS-S	1	.404**	.150**	.182**	.267**	.320**	**.601****	-.108**	-.253**	**.860****	**.510****	-.212**
	F2 CRS-S		1	.266**	.014	.221**	.110**	.394**	-.156**	-.280**	**.814****	.349**	-.217**
	F11 PF			1	-.038	.183**	.052*	.151**	-.142**	-.370**	.244**	**.508****	-.301**
	F12 PF				1	.248**	.170**	.171**	-.107**	.075**	.123**	**.561****	-.047*
	F13 PF					1	.308**	.374**	-.197**	-.243**	.293**	**.619****	-.067**
	F21 PF						1	.413**	-.099**	-.147**	.265**	**.584****	-.159**
	F22 PF							1	-.168**	-.247**	**.599****	**.701****	-.265**
	F1 CP								1	.133**	-.154**	-.228**	**.877****
	F2 CP									1	-.316**	-.303**	**.593****
	CRS-S-T										1	**.516****	-.283**
	PF-T											1	-.338**
S&D	F1 CRS-S	1	.497**	-.052	.131*	.007	.139*	.481**	-.113	-.076	**.875****	.302**	-.137**
	F2 CRS-S	.497**	1	.049	-.021	.033	.125	.435**	-.077	-.051	**.855****	.274**	.093**
	F11 PF	-.052	.049	1	-.008	.280**	.098	.112	-.102	-.443**	-.004	**.517****	-.282**
	F12 PF	.131*	-.021	-.008	1	.294**	.197**	.073	-.145*	-.077	.067	**.519****	-.136*
	F13 PF	.007	.033	.280**	.294**	1	.376**	.137*	-.261**	-.366**	.023	**.583****	-.372**
	F21 PF	.139*	.125	.098	.197**	.376**	1	.333**	-.177**	-.295**	.152*	**.621****	-.292**
	F22 PF	.481**	.435**	.112	.073	.137*	.333**	1	-.176*	-.209**	**.522****	**.684****	-.241**
	F1 CP	-.113	-.077	-.102	-.145*	-.261**	-.177**	-.176*	1	.274**	-.110	-.310**	**.833****
	F2 CP	-.076	-.051	-.443**	-.077	-.366**	-.295**	-.209**	.274**	1	-.074	-.456**	**.694****
	CRS-S-T	.875**	.855**	-.004	.067	.023	.152*	.522**	-.110	-.074	1	.328**	-.134**
	PF-T	.302**	.274**	.517**	.519**	.583**	.621**	.684**	-.310**	-.456**	.328**	1	-.452**

Legend. CRS-S, PF y CP, is the abbreviated way of referring to CRS-S$_{Eg\text{-}S\&D}$, PAFAS, and CAPES in this table (for reasons of space); F1 and F2 in CRS-S are *Coparentalidad Positiva* and *Percepción Negativa de la Coparentalidad*, respct., F11, F12, F13, F21, and F22 in PAFAS are *Family adjustment*, *Positive encouragement*, *Parent-child relationship*, *Parental adjustment*, and *Family adjustment*, respct.; F1 and F2 in CAPES are *Child's competencies*, and *Behavioural and emotional problems*, respct.; CRS-S-T, PF-T and CP-T= Score on the total of the respective scales. All scores were transformed as indicated in the text (in PAFAS, each factor could achieve a maximum of 0.20, and in CRS-SEg-S&D and CAPES, each factor could achieve a maximum of 0.5, such that the total sum of each scale can reach a maximum value of 1); **,*= p<0.01 and p=<0.05, respct.

Mukaka M. M. (2012). Statistics corner: A guide to appropriate use of correlation coefficient in medical research. *Malawi medical journal: the journal of Medical Association of Malawi*, *24*(3), 69-71

Table S7. In the Female sample. Empirical correlations between the factors of the CRS questionnaire adapted and validated for the population of Spanish parents, CRS-S$_{Eg\text{-}S\&D}$, and the factors of the PAFAS (5 factors) and CAPES (two factors) questionnaires. Correlations between the total scores of the scales are also shown. The results are presented segmented according to marital status, combining men and women in the estimation. Correlations greater than .50 stand out (Mukaka, 2012)

		F1 CRS-S	F2 CRS-S	F11 PF	F12 PF	F13 PF	F21 PF	F22 PF	F1 CP	F2 CP	CRS-S-T	PF-T	CP-T
Eg	F1 CRS-S	1	.420**	.151**	.174**	.268**	.306**	**.612****	-.110**	-.256**	**.871****	**.507****	-.219**
	F2 CRS-S		1	.280**	.002	.229**	.138**	.406**	-.158**	-.273**	**.812****	.360**	-.268**
	F11 PF			1	-.012	.175**	.067**	.152**	-.145**	-.365**	.248**	**.520****	-.303**
	F12 PF				1	.269**	.157**	.151**	-.110**	.066**	.113**	**.562****	-.051*
	F13 PF					1	.305**	.396**	-.193**	-.254**	.296**	**.625****	-.269**
	F21 PF						1	.414**	-.095**	-.165**	.272**	**.583****	-.170**
	F22 PF							1	-.163**	-.251**	**.610****	**.698****	-.264**
	F1 CP								1	.128**	-.155**	-.223**	**.875****
	F2 CP									1	-.311**	-.313**	**.592****
	CRS-S-T										1	**.519****	-.284**
	PF-T											1	-.338**
S&D	F1 CRS-S	1	**.525****	-.037	.099	.000	.158*	.494**	-.077	-.139	**.883****	.294**	-.128
	F2 CRS-S		1	-.021	.000	.022	.159*	.436**	-.056	-.057	**.864****	.251**	-.071
	F11 PF			1	.029	.291**	.121	.115	-.104	-.449**	-.034	**.537****	-.285**
	F12 PF				1	.320**	.221**	.069	-.091	-.072	.059	**.533****	-.079
	F13 PF					1	.423**	.160*	-.288**	-.412**	.012	**.618****	-.414**
	F21 PF						1	.312**	-.196**	-.319**	.181*	**.633****	-.308**
	F22 PF							1	-.163*	-.252**	**.523****	**.670****	-.239**
	F1 CP								1	.281**	-.076	-.303**	**.886****
	F2 CP									1	-.114	-.492**	**.694****
	CRS-S-T										1	.305**	-.115
	PF-T											1	-.448**

Legend. Legend. Se destacan algunas correlaciones >.40 debido a la notoria diferencia en magnitud observada en las submuestras de hombres y mujeres. Para el resto, see Table S6

Table S8. In the Male sample. Empirical correlations between the factors of the CRS questionnaire adapted and validated for the population of Spanish parents, CRS-S$_{Eg\text{-}S\&D}$, and the factors of the PAFAS (5 factors) and CAPES (two factors) questionnaires. Correlations between the total scores of the scales are also shown. The results are presented segmented according to marital status, combining men and women in the estimation. Correlations greater than .50 stand out (Mukaka, 2012)

		F1 CRS-S	F2 CRS-S	F11 PF	F12 PF	F13 PF	F21 PF	F22 PF	F1 CP	F2 CP	CRS-S-T	PF-T	CP-T
Eg	F1 CRS-S	1	.346**	.134**	.269**	.289**	.411**	**.555****	-.101*	-.224**	**.802****	**.553****	-.176**
	F2 CRS-S		1	.225**	.053	.189**	-.002	.344**	-.147**	-.319**	**.838****	.300**	-.280**
	F11 PF			1	-.113*	.229**	.001	.150**	-.136**	-.380**	.221**	.475**	-.294**
	F12 PF				1	.159**	.219**	.264**	-.093	.081	.190**	**.556****	-.042
	F13 PF					1	.321**	.291**	-.212**	-.224**	.289**	**.602****	-.268**
	F21 PF						1	.410**	-.112*	-.076	.237**	**.589****	-.117*
	F22 PF							1	-.189**	-.229**	**.542****	**.720****	-.269**
	F1 CP								1	.165**	-.152**	-.253**	**.890****
	F2 CP									1	-.334**	-.272**	**.597****
	CRS-S-T										1	**.513****	-.280**
	PF-T											1	-.344**
S&D	F1 CRS-S	1	.393**	-.165	.244	.029	.040	.435**	-.284	.264	**.846****	.327*	-.179
	F2 CRS-S		1	.296*	-.082	.072	-.060	.432**	-.167	.039	**.823****	.331*	-.160
	F11 PF			1	-.120	.215	-.095	.087	-.056	-.254	.069	.359*	-.128
	F12 PF				1	.208	.111	.089	-.412**	-.172	.103	**.508****	-.461**
	F13 PF					1	.083	.024	-.102	-.108	.059	.357*	-.143
	F21 PF						1	.436**	-.028	-.095	-.010	**.540****	-.131
	F22 PF							1	-.241	-.009	**.521****	**.761****	-.269
	F1 CP								1	.184	-.277	-.350*	**.873****
	F2 CP									1	.184	-.181	**.641****
	CRS-S-T										1	.397**	-.207
	PF-T											1	-.434**

Legend. Legend. Se destacan algunas correlaciones >.40 debido a la notoria diferencia en magnitud observada en las submuestras de hombres y mujeres. Para el resto, see Table S6

Section S4. This section shows the information referred to in the third point of the article in section **4. Discussion and Conclusions.** This information is entered in the paragraphs that appear in the text of the article and are highlighted in red for a better location.

Third. Experts in the method have shown that a good model fit does not prove that the model is theoretically sound [64,80], that larger samples produce more precise solutions [98,99], that replication (perform the analysis using Cross-validation and by evaluating the difference between factor loadings) avoids overfitting of the models and adds value to the result of the Factor Analysis [38,96], and that the EFA should always be a prior step to the CFA [42,100].

In this research, the proportion of Eg parents is much higher than that of S&D parents, and in each marital status, women are represented in a greater proportion than men. This also happens in the three reference investigations. The percentage of women is 70%, 63.40% and 56.9%, respectively in Costa et al. [34], Dumitriu et al. [36], and Favez et al. [35], and the percentage of Eg is 80% in Costa et al. [34], and 69% in Dumitriu et al. [36], (Favez et al. [35] do not indicate this detail). However, this research distances itself from the three reference investigations in critical methodological aspects, which we summarize in two points.

One, the ratio between the sample size and the number of items is very large (41.3:1), being far removed from the research carried out by Costa et al. [34], Favez et al. [35] and Dumitriu et al. [36], which was 19.20:1, 11.4:1 and 14.4:1, respectively (this same occurs in research with parents of samples with unique characteristics as can see in **Section S4 of the Supplementary material).** This same trend occurs in research carried out with parents of samples that had unique characteristics [Lamela et al., 2016 (12.86:1); Pinto et al., 2019 (26.16:1); Lee et al., 2020 (12.14:1); Lamela et al., 2018 (11.65:1), and Leal et al., 2022 (4.7:1 and 5.6:1 in the two subsamples)].

Two, in none of the three, is the analysis carried out through cross-validation, and in none is the value given to the descriptive analysis of the items to examine their suitability to be part of the scale (it is only done to decide on the estimation method). In none of them was an EFA previously carried out, and in all three, the model of Feinberg et al. was tested directly using CFA. [26] of 7 factors.

Favez et al. [35], despite observing that items 13, 16, 21, and 22 were strongly skewed and had a very high kurtosis, despite watching that they had a mean value very close to the lowest response value for the item, specifically (0.57, 0.51, 0.63 and 0.75 in the total sample, respectively) and that their SD was less than 1, they decided not to dispense with any item and use robust tests.

Costa et al. [34] and Dumitriu et al. [36] considered reducing the number of items in the scale was appropriate. Costa et al. [34] eliminated 4 items (items 13, 28, 5, and 20), and Dumitriu et al. [36] eliminated 7 items (items 6, 7, 8, 28, 29, 5, and 20). Both it was done based on the factor loading or R2 observed in the CFA solution. Despite the eliminated items (only Costa et al. [34] decided to eliminate the Division of Labor factor), neither of the two investigations considered that the dimensionality is different due to removing items. In this research, 10 items have been eliminated, including the 4 eliminated by Costa et al. [34] and 4 (of 7) eliminated by Dumitriu et al. [36]. **The Sumplementary material (Section S4)** describes the items destroyed in the investigations with singular samples. In research conducted with singular samples items have also been removed and this has also been done in the course of conducting the CFA, Lamela et al, (2016) removed items 5 and 20, Pinto et al, (2019) items 30, 31, 32, 33, 34 and 35; Lamela et al, (2018) items 5, 20, 6, 7 and 28, and Leal et al, (2022) items 5, 12, 15, 17 and 18).

Furthermore, Favez et al. [35] and Dumitriu et al. [36] conclude that the 7-factor Feinberg model fits their data, and Costa et al. [34] conclude that the model fits with six factors. However, in the three cases the model is fitted tangentially (Costa el al. [34]: [χ^2/gl = 4.69; CFI =0 .90; GFI = 0.85, RMSEA = 0.07], Dumitriu et al. [36] [χ^2/gl = 3.90, y RMSEA =0 .076] y Favez et al. [35] [χ^2/gl = 2.94; CFI = 0.863; RMSEA = 0.07; y SRMR=0.078], and although experts emphasize that a well-fitted model is not indicative that the model is valid, in none of these three investigations was it considered testing a different model, based, for example, on the high correlation between some of the factors, an aspect that can be seen in these three investigations, and in the investigations carried out with singular samples (see Section 5 in the supplementary material).

Section S5. Identification data of the participation of educational centers

We start from the premise that in any country, the native population dominates. However, today, we know that the ethnic, cultural, and religious mix already significantly represents all populations due to the mobility caused by work, political exile, etc.

The data have been collected in public, private, and subsidized rural and urban schools in the four provinces of Galicia, assuming that the sample of parents is representative of the population of parents throughout Spain. Data has also been collected in Melilla. This decision was made because a significant proportion of people from North Africa live in Spain. Still, in Galicia specifically, this proportion is lower than that of other Spanish communities. However, the proportion of people from Europe or South America is similar to that of different autonomous communities in Spain.

The sample comprises 3,155 parents of children attending 73 schools in the four provinces of Galicia and Melilla. Although the school to which the children of 80 participants (2.53%) go is unknown, the most significant volume of responses, 1241 (40%), is collected in 9 schools (12.32%) where more than 100 parents participate in each one. The smallest volume of responses, 64 (2.08%), is collected in 22 schools (30.13%) where less than 10 parents participate. Between these two, there are schools where between 50-100 parents participate (14 schools, 19.17%) and where between 10-50 parents participate (28 schools, 38.56%), collecting the responses of 982 (31.93%) and 788 (25.62%) of the parents respectively. Table 1 shows graphically and tabularly the distribution of school participation.

Table S9. Exhaustive distribution of participation in schools is shown in the graph (Left), and distribution is ordered in participation intervals in a tabular way (Right).

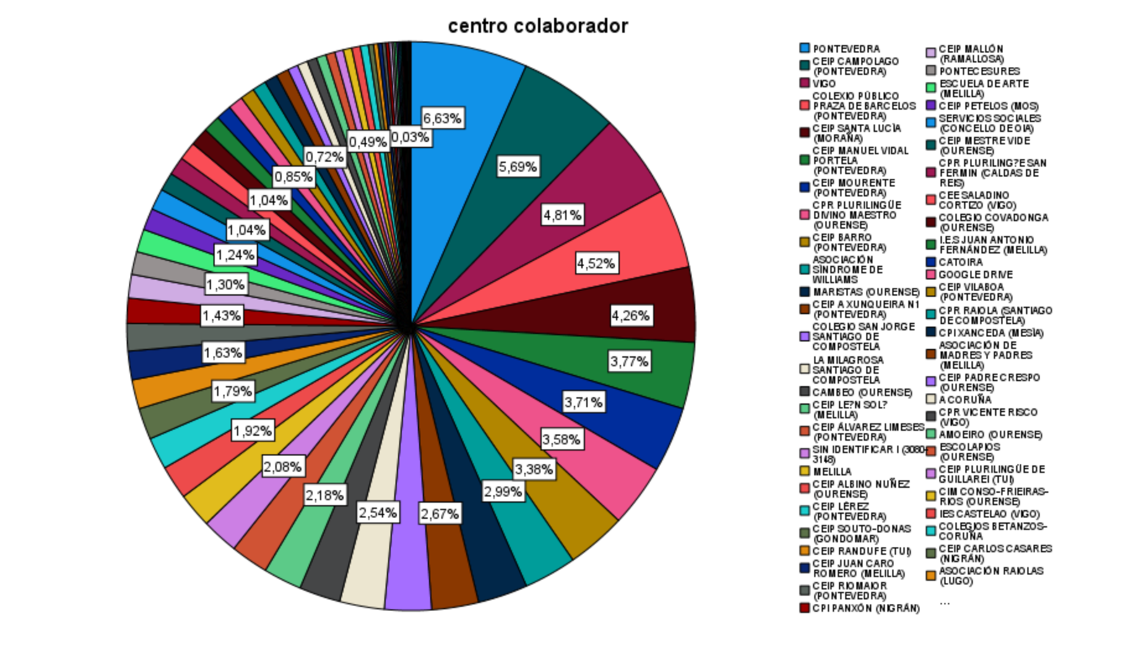

	Participation or data collection	Schools	Parents
Participation in schools	< 10 parents	22 (30.13%)	64 (2.08%)
	10-50 parents	28 (38.56%)	788 (25.62%)
	51-100 parents	14 (19.17%)	982 (31.93%)
	>100-204 parents	9 (12.32%)	1,241 (40.35%)
	[1]Total	73	3,075

Legend. [1]= The school of the children of the 80 people who submitted the answer booklet is unknown (2.53%).

Regarding the country of birth, 89.99% (n=2,686) are Spanish (see table to examine the participants who are from Galicia and Melilla), 4.46% (n=133) are from a country in South America, 3.18% (n=95) are from Western Europe (central Europe), far from the latter, 2.04% (n=61) are African (1.84% from Morocco). Testimonially, less than .20% are from Eastern Europe, USA-Canada, and China.
